# Supplementary figures and images for: Excitatory-inhibitory homeostasis and bifurcation control in the Wilson-Cowan model of cortical dynamics
Source: PLoS Comput Biol. 2025 Jan 6;21(1):e1012723. doi: 10.1371/journal.pcbi.1012723 (PMC11737862; doi:10.1371/journal.pcbi.1012723)

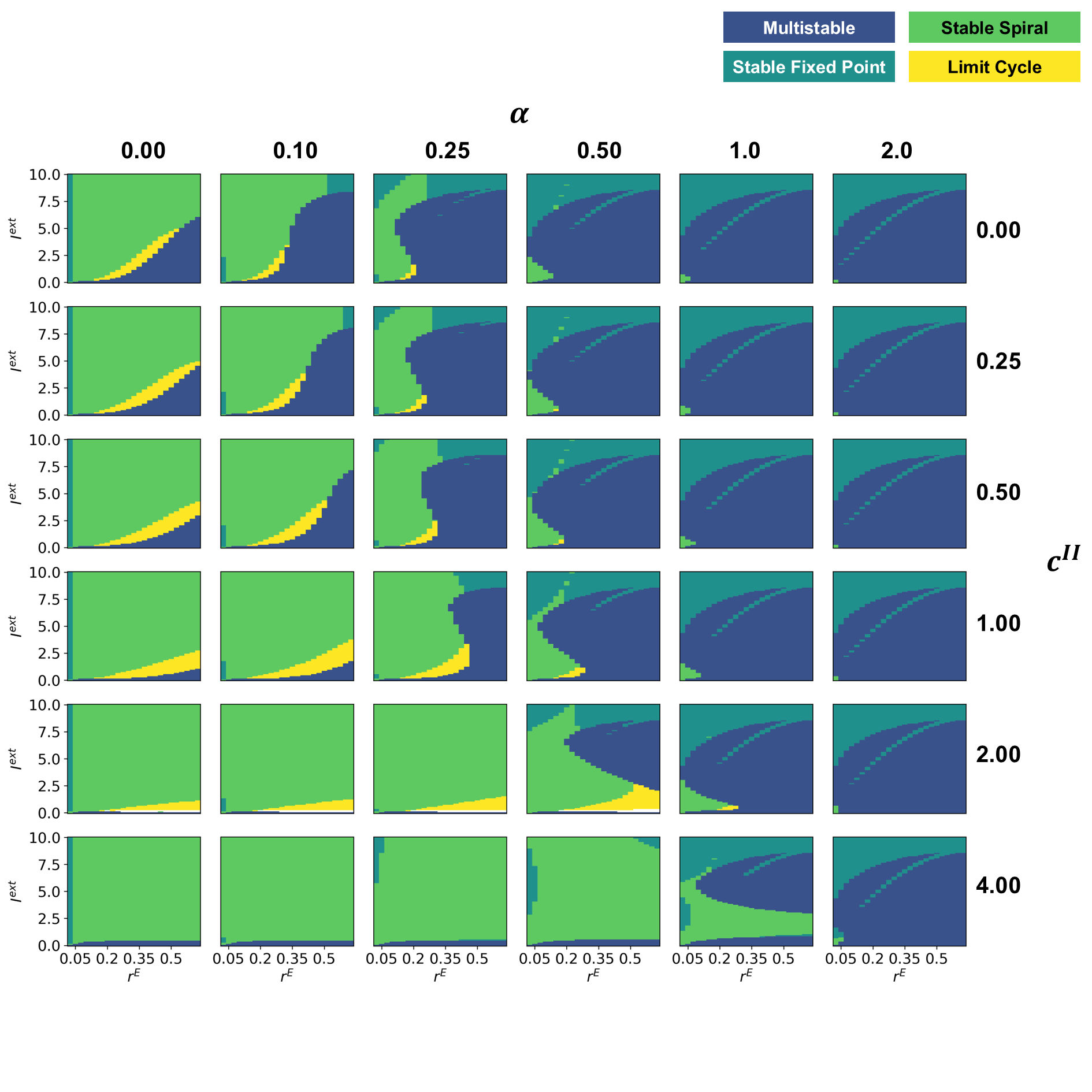

Supplement: S1 Fig — Circuit dynamics of models under homeostasis of GE, cEI, μE, and σE with different combinations of external input Iext and fixed point rE. Colors represent the result of linear stability analysis for each combination of parameters, as described by the legend in the top-right. In each sub-plot, we present the results of this analysis for models with a different combination of cII, the strength of self-inhibition, and α, representing the relative strength of the external input to the inhibitory population, compared to Iext (input to the excitatory neural mass). (TIF) [file pcbi.1012723.s001.tif]

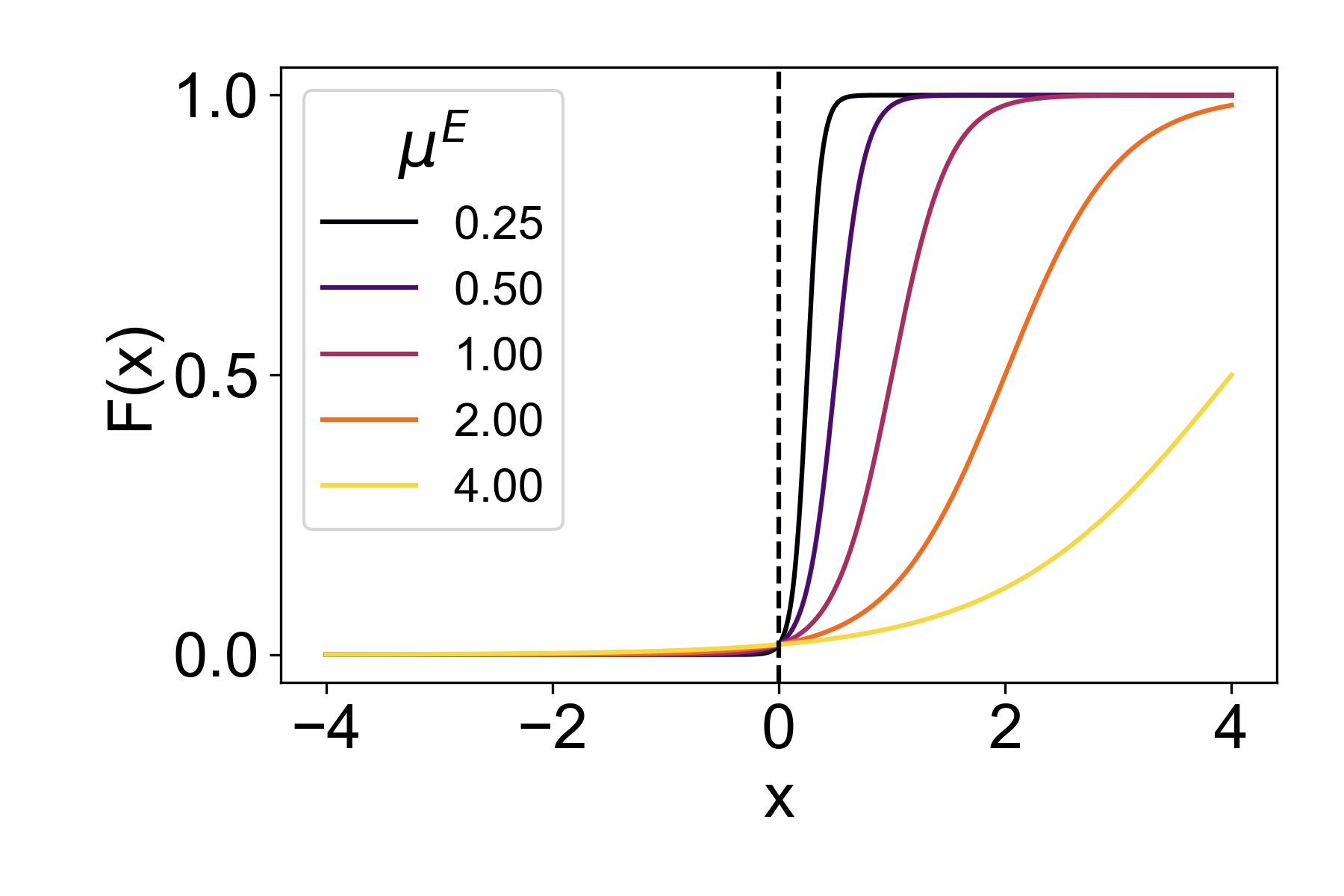

Supplement: S2 Fig — Note that the value of F(0) is always the same as long as σE = KμE. (TIF) [file pcbi.1012723.s002.tif]

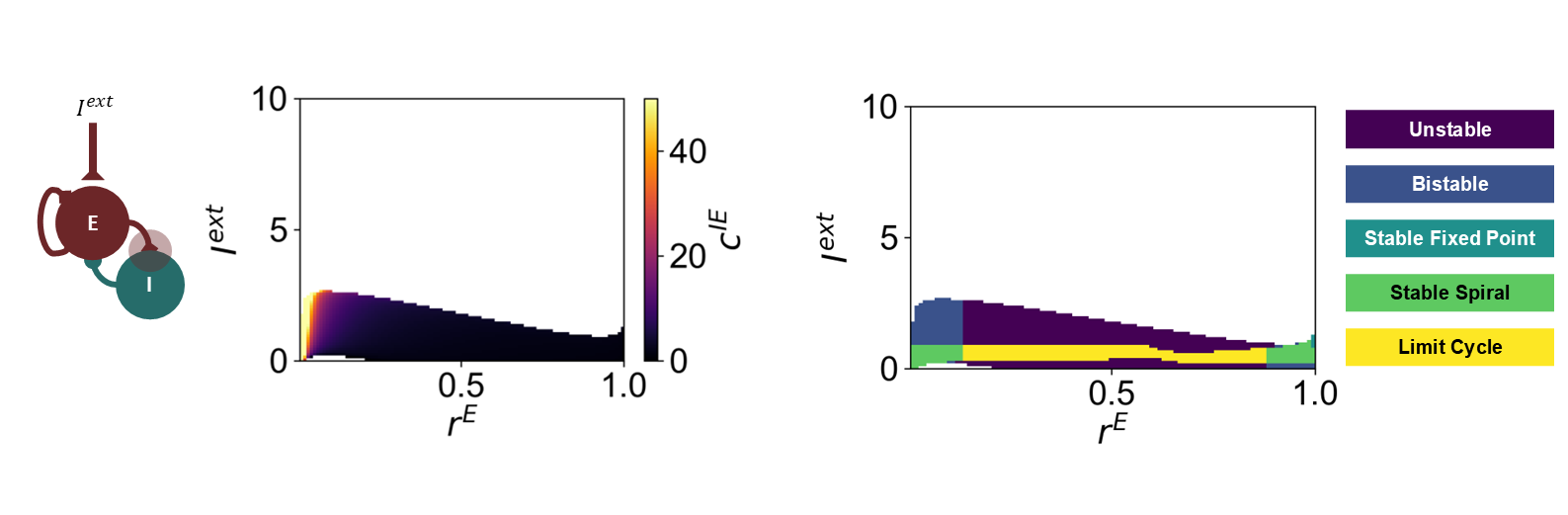

Supplement: S3 Fig — (Left) Homeostatic value of cIE and (Right) corresponding system behavior for different combinations of rfixedE and Iext. (TIF) [file pcbi.1012723.s003.tif]

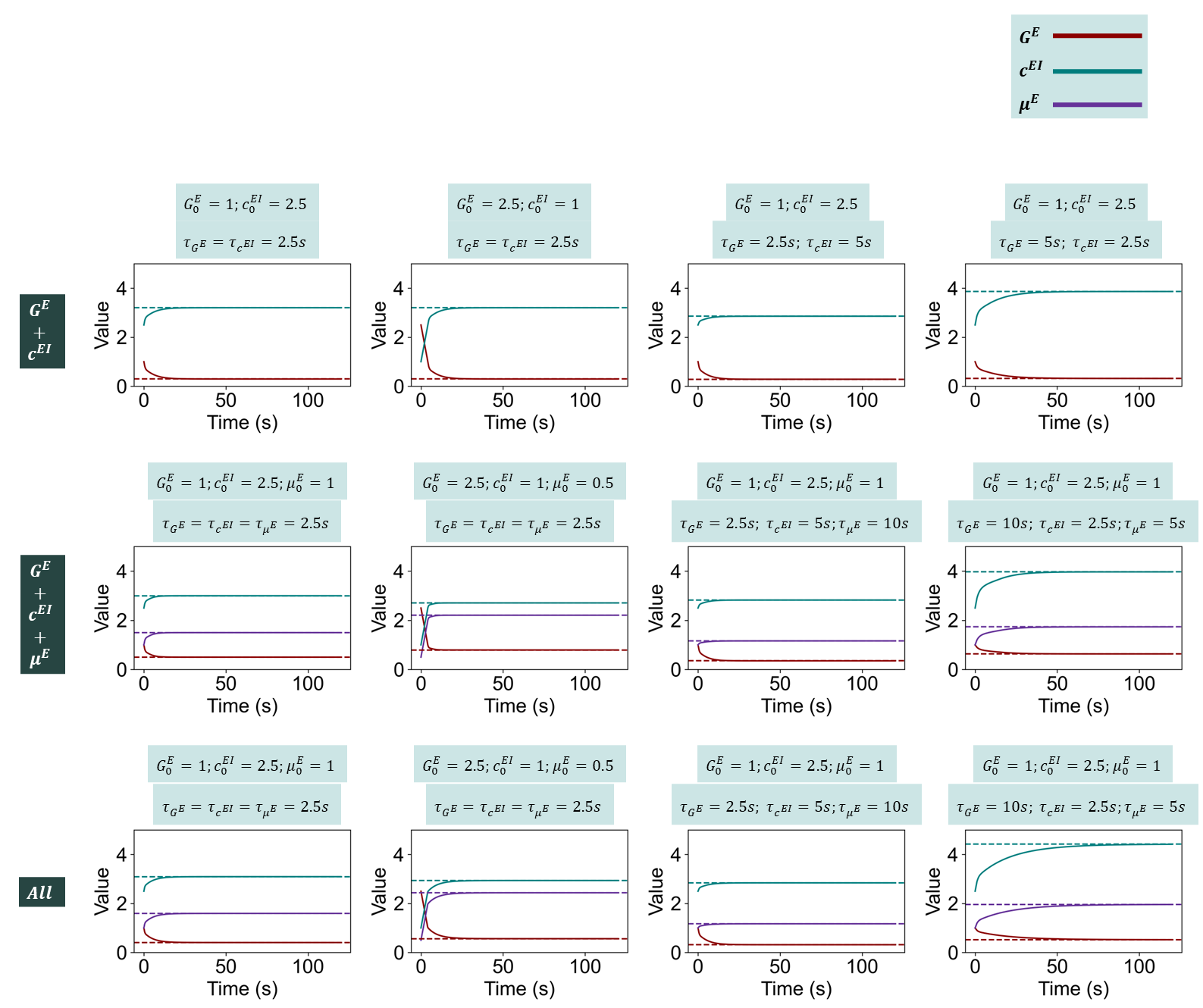

Supplement: S4 Fig — Solid lines represent the model parameters at any given point in time. Conversely, dashed lines represent the predicted values. For all simulations, we used Iext = 2 and ρ = 0.1, and the equations were integrated using the Euler method with a time step of 0.2 ms. (TIF) [file pcbi.1012723.s004.tif]

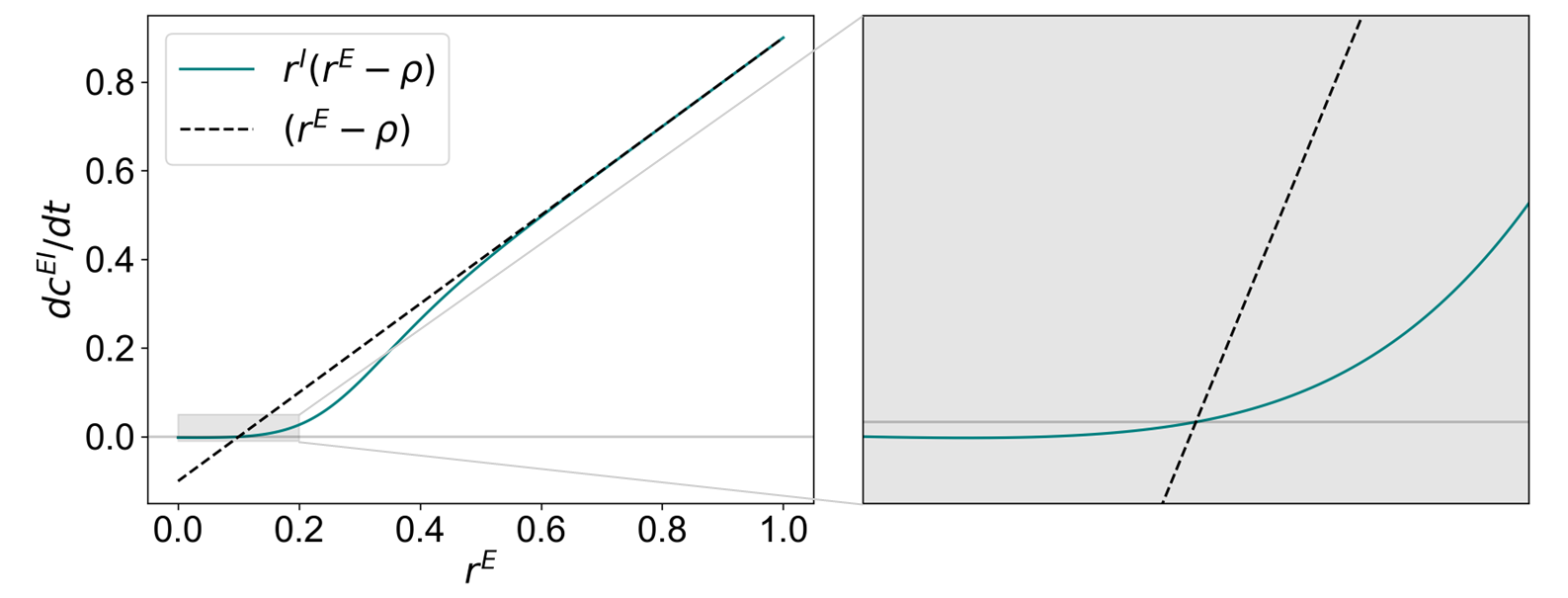

Supplement: S5 Fig — We present two cases where dcEIdt=rI(rE-ρ) (solid blue line) or dcEIdt=rE-ρ (dashed black line). For illustration purposes, we consider that τhomeo = 1s and ρ = 0.1. (TIF) [file pcbi.1012723.s005.tif]

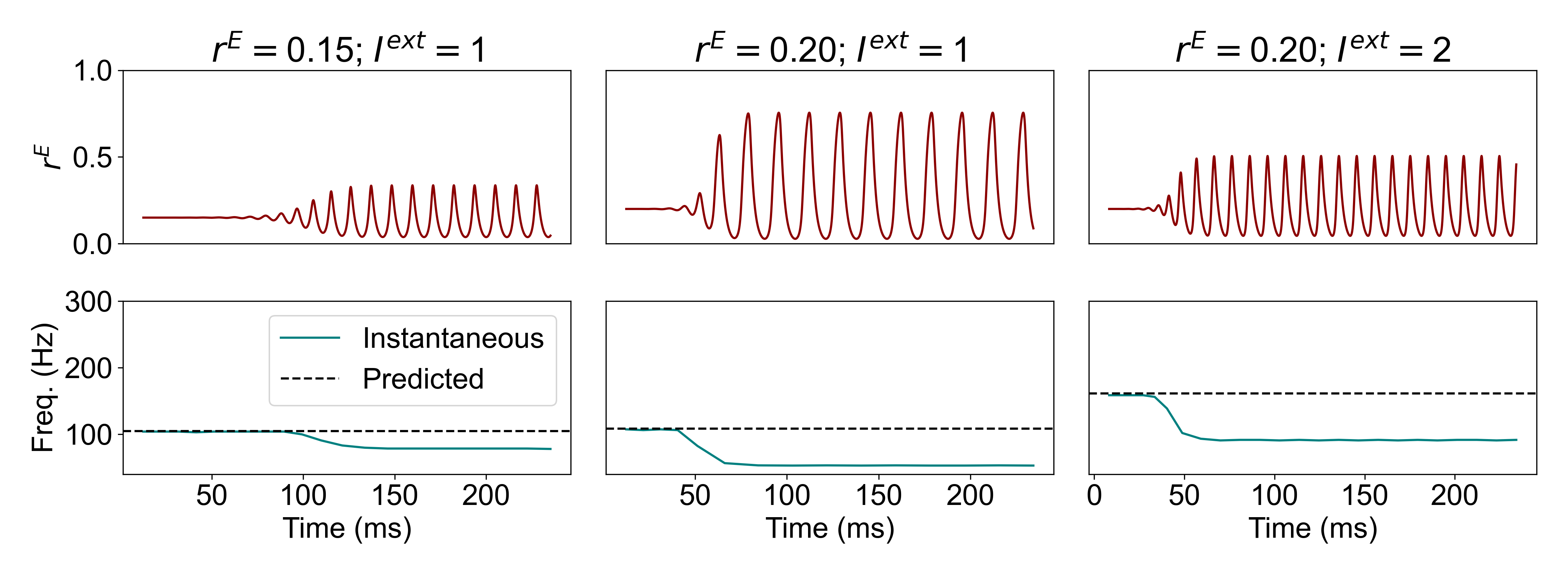

Supplement: S6 Fig — The black dashed line represents the analytical prediction for the frequency of oscillation around the fixed point. (TIF) [file pcbi.1012723.s006.tif]

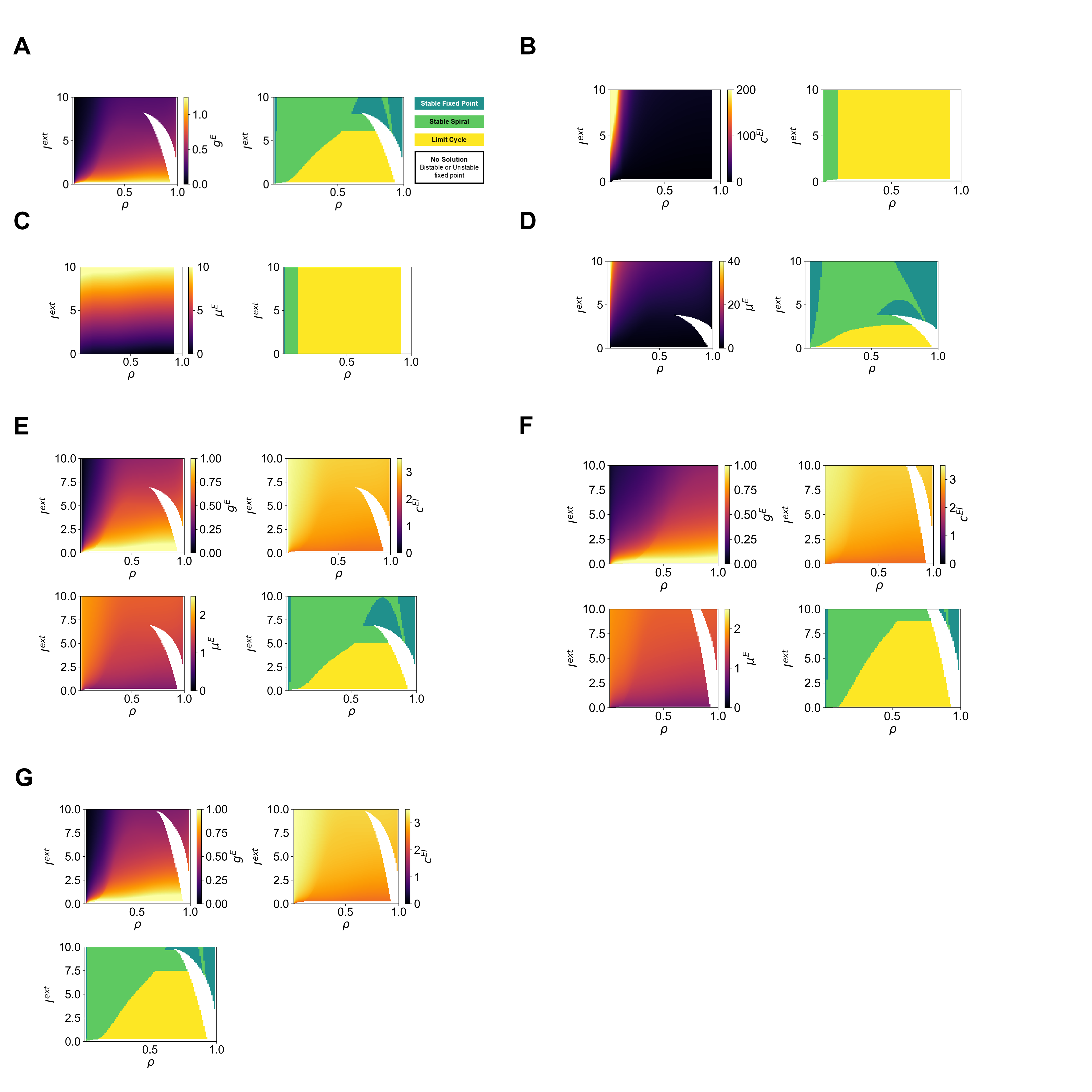

Supplement: S7 Fig — a) Homeostatic value of GE (Left) and model dynamics (Right) in the model with homeostasis of GE. b) Homeostatic value of cEI (Left) and model dynamics (Right) in the model with homeostasis of cEI. c) Homeostatic value of μE (Left) and model dynamics (Right) in the model with homeostasis of μE. d) Homeostatic value of μE (Left) and model dynamics (Right) in the model with homeostasis of μE and σE. For all values of μE, σE = KμE e) Homeostatic value of GE (Top Left), cEI (Top Right) and model dynamics (Bottom) in the model with homeostasis of GE and cEI. f) Homeostatic value of GE (Top Left), cEI (Top Right), μE (Bottom Left) and model dynamics (Bottom Right) in the model with homeostasis of GE, cEI and μE. g) Homeostatic value of GE (Top Left), cEI (Top Right), μE (Bottom Left) and model dynamics (Bottom Right) in the model with homeostasis of GE, cEI, μE and σE. For all values, σE = KμE. (TIF) [file pcbi.1012723.s007.tif]

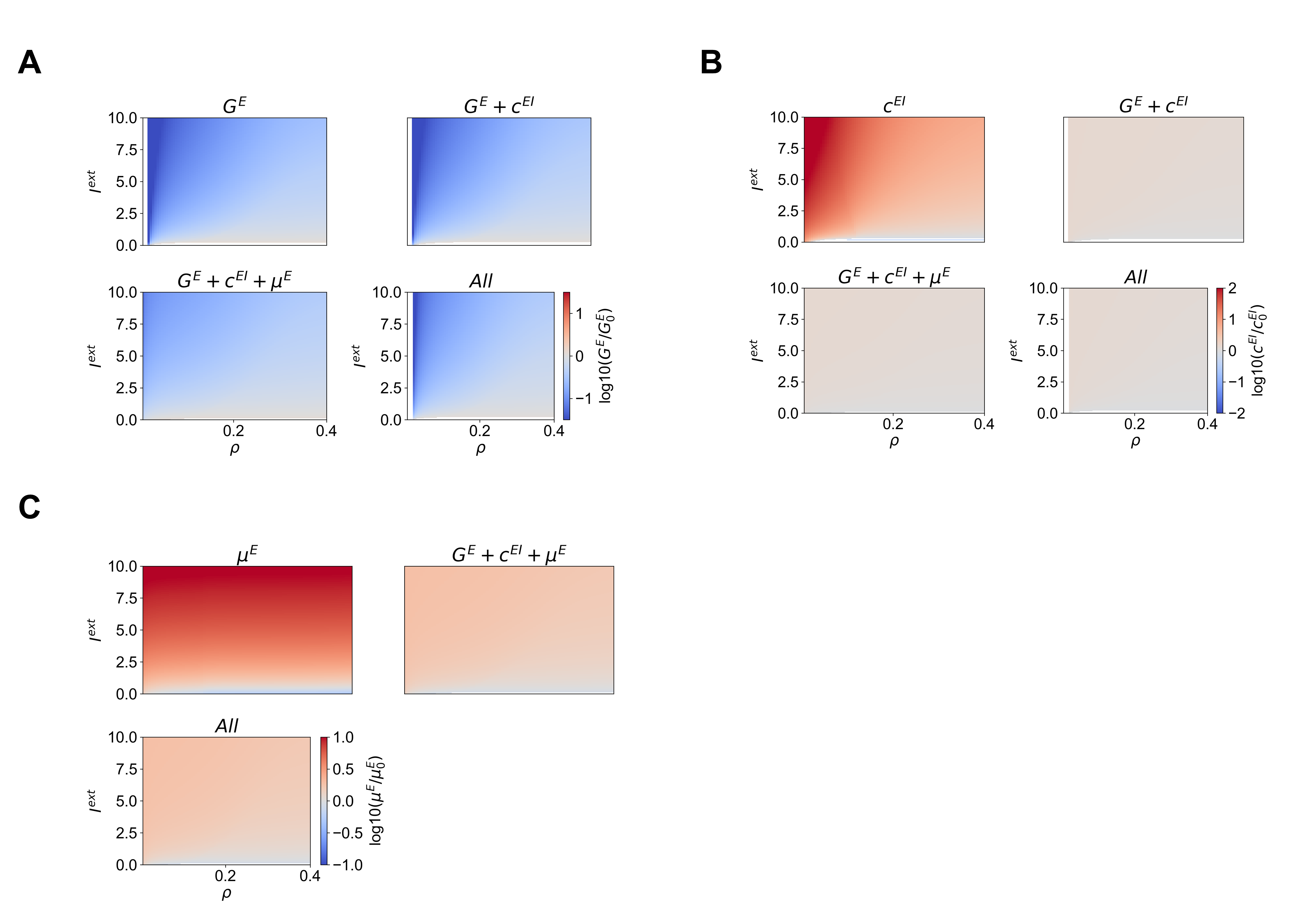

Supplement: S8 Fig — a) Value of GE compared to the default for different modes of homeostasis. Plots show the log difference between GE and default G0E=1 for different combinations of ρ and Iext in models with homeostasis of GE (Top Left), GE + cEI (Top Right), GE + cEI + μE (Bottom Left) and GE + cEI + μE + σE (Bottom Right) b) Value of cEI compared to the default for different modes of homeostasis. Plots show the log difference between cEI and default c0EI=2.5 for different combinations of ρ and Iext in models with homeostasis of GE (Top Left), GE + cEI (Top Right), GE + cEI + μE (Bottom Left) and GE + cEI + μE + σE (Bottom Right) c) Value of μE compared to the default for different modes of homeostasis. Plots show the log difference between μE and default μ0E=1 for different combinations of ρ and Iext in models with homeostasis of GE (Top Left), GE + cEI (Top Right), GE + cEI + μE (Bottom Left) and GE + cEI + μE + σE (Bottom Right). (TIF) [file pcbi.1012723.s008.tif]
