# Supplementary material for: Excitatory-inhibitory homeostasis and bifurcation control in the Wilson-Cowan model of cortical dynamics
Source: PLoS Comput Biol. 2025 Jan 6;21(1):e1012723. doi: 10.1371/journal.pcbi.1012723 (PMC11737862; doi:10.1371/journal.pcbi.1012723)
Supplement: S1 Appendix — (PDF) [file pcbi.1012723.s009.pdf]

S1 Appendix Effects of E-I Homeostasis in Models with Self-Inhibition and Non-Zero Input to the Inhibitory Population

In the main text, we set both c^{II} and α to zero, in order to simplify the analytical analysis of the model but, most importantly, to ensure that the model exhibits a Hopf-bifurcation between a fixed point and a limit-cycle [45]. This would correspond to the transition between the green and yellow areas in the upper-left panel of S1 Fig, where c^{II} and α are set to 0. However, empirical studies suggest that self-inhibitory coupling exists not only between interneuron types, but also between fast-spiking interneurons, [51, 81], which are the focus of our study. In addition, while we assume that there is no external input to the inhibitory population, recent studies show that cortical interneurons not only receive long-range inputs in the cortex, but that these originate from the same areas as the inputs to their pyramidal counterparts [97, 98]. Therefore, a more biophysically realistic model should include both self-inhibition and an external input to the inhibitory population. This can be easily implemented in the Wilson-Cowan model as follows:

$$\begin{aligned}\tau^E \frac{r^E(t)}{dt} &= -r^E(t) + F^E\left(G^E c^{EE} r^E(t) - c^{EI} r^I(t) + G^E I^{ext}(t)\right) \\ \tau^I \frac{r^I(t)}{dt} &= -r^I(t) + F^I\left(c^{IE} r^E(t) + c^{II} r^I + \alpha I^{ext}\right)\end{aligned} \tag{20}$$

where the external input to the inhibitory population is written as αI^{ext}, representing a scaled version of the input to the excitatory neural mass, reflecting the fact that both inputs come from largely overlapping sources [97, 98].

With this form of the model, the r^E and r^I nullclines equations are, respectively:

$$r^I = \frac{\sigma^E}{c^{EI}} log\left(\frac{1-r^E}{r^E}\right) + \frac{G^E c^{EE} r^E + G^E I^{ext} - \mu^E}{c^{EI}} \tag{21}$$

$$r^E = -\frac{\sigma^I}{c^{IE}} log\left(\frac{1-r^I}{r^I}\right) + \frac{c^{II} r^I - \alpha I^{ext} - \mu^I}{c^{IE}} \tag{22}$$

In this case, since the r^I nullcine (Eq 22) cannot be re-ordered as an expression of $r^I = ...$, the homeostatic parameters corresponding to the fixed point need to be computed differently. In this context, it is relevant to note that the r^I nullcline does not depend on any of the parameters that can be modulated by E-I homeostasis (i.e. G^E, c^{EI}, μ^E and σ^E). Therefore, given a combination of r^E_{fixed} and I^{ext}, Eq 22 can be used to compute r^{fixed}, which can then substitute r^I in Eq 21 to obtain the homeostatic value of any given parameter.

Here, to analyze how self-inhibition and a non-zero input to the inhibitory population shape the effect of E-I homeostasis on model dynamics, we focus on the combined homeostasis of G^E, c^{EI}, μ^E and σ^E as an example. Therefore, Eq 23 can be updated as:

$$\begin{aligned}\mu^E &= \frac{(c_0^{EI} - \mu_0^E) r^I_{fixed} - (G_0^E + \mu_0^E)(c^{EE} r^E + I^{ext})}{K log\left(\frac{1-r^E}{r^E}\right) - 1 - r^I_{fixed} - c^{EE} r^E - I^{ext}} \\ \sigma^E &= K\mu^E \\ G^E &= G_0^E - \mu^E + \mu_0^E \\ c^{EI} &= c_0^{EI} + \mu^E - \mu_0^E\end{aligned} \tag{23}$$

where r^{fixed} can be computed from Eq 22.

That said, we use linear-stability analysis to study the dynamics of models with different levels of c^{II} and α and the homeostasis of G^E, c^{EI}, μ^E and σ^E (S1 Fig). We

aim to investigate to which extent such models can still display a Hopf bifurcation between the stable spiral and limit-cycle regime, which is essential to establish a relationship between E-I homeostasis and edge-of-bifurcation dynamics. The results of this analysis demonstrate that the Hopf bifurcation can still be observed in models with self-inhibition ($c^{II} > 0$) and input to the inhibitory population ($\alpha > 0$). Focusing on the effects of c^{II}, our results demonstrate that, as it is increased, the bifurcation is increased to higher values of r^E (S1 Fig). However, when this value is increased beyond a certain point (in this case, $c^{II} > 2$), the circuit loses the ability to engage in sustained oscillations while maintaining the homeostasis of firing rates, as evidenced by the disappearance of the yellow area in the parameter space. When analyzing the effects of increasing the input to the inhibitory population, our results suggest that the main effect is an extension of the region with multistable dynamics, in line with the original results of [44], leading to the progressive disappearance of the limit-cycle region in the parameter space. However, this effect can be counteracted by increasing self-inhibition. For example, while the model with $\alpha = 0.5$ does not show any homeostatic set-point with limit-cycle dynamics when $c^{II} = 0$, the limit-cycle area of the parameter is greatly expanded when c^{II} is set to 2 (S1 Fig).

In summary, these results suggest that the Wilson-Cowan model with homeostatic regulation of firing rates can still exhibit a Hopf-bifurcation when self-inhibition and inputs to the inhibitory population are accounted for. Therefore, we argue that the main conclusions of our study regarding the impact of E-I homeostasis on bifurcation control would still be valid in more biophysically realistic models of the human cortex, provided that both quantities remain within certain bounds. In this regard, empirical studies suggest that I-I interactions are indeed weaker than I-E connectivity in cortical networks [81, 99], suggesting that c^{II} should have a lower magnitude than c^{IE} or c^{EI}. In addition, studies also indicate lower inputs to inhibitory neurons than to pyramidal cells [81], indicating that α should at least be lower than 1. Therefore, we argue that, under these assumptions, the main conclusions of our analysis in the main text are likely to be transferable to more realistic models with self-inhibition and external inputs to the inhibitory population.
