# Supplementary material for: Excitatory-inhibitory homeostasis and bifurcation control in the Wilson-Cowan model of cortical dynamics
Source: PLoS Comput Biol. 2025 Jan 6;21(1):e1012723. doi: 10.1371/journal.pcbi.1012723 (PMC11737862; doi:10.1371/journal.pcbi.1012723)
Supplement: S2 Appendix — (PDF) [file pcbi.1012723.s010.pdf]

S2 Appendix Derivation of Nullclines and Fixed Point Expression

The expression for the r^E nullcline can be obtained from Eqs 1 and 2 through the following chain of operations:

$$
\begin{aligned}
0 &= \frac{dr^E}{dt} \\
0 &= -r^E + F^E\left(G^E c^{EE} r^E - c^{EI} r^I + G^E I^{ext}\right) \\
r^E &= \frac{1}{1 + exp(-(G^E c^{EE} r^E - c^{EI} r^I + G^E I^{ext} - \mu^E)/\sigma^E)} \\
\frac{1}{r^E} &= 1 + exp(-(G^E c^{EE} r^E - c^{EI} r^I + G^E I^{ext} - \mu^E)/\sigma^E) \\
\frac{1}{r^E} - 1 &= exp((c^{EI} r^I)/\sigma^E) exp(-(G^E c^{EE} r^E + G^E I^{ext} - \mu^E)/\sigma^E)
\end{aligned}
\tag{24}
$$

Then, we isolate the r^I term on one side of the equation to obtain the nullcline, expressing r^I as a function of r^E and the model parameters:

$$
\begin{aligned}
exp((c^{EI} r^I)/\sigma^E) &= (\frac{1 - r^E}{r^E}) exp((G^E c^{EE} r^E + G^E I^{ext} - \mu^E)/\sigma^E) \\
\frac{c^{EI} r^I}{\sigma^E} &= log\left(\frac{1 - r^E}{r^E}\right) + \frac{G^E c^{EE} r^E + G^E I^{ext} - \mu^E}{\sigma^E} \\
r^I &= \frac{\sigma^E}{c^{EI}} log\left(\frac{1 - r^E}{r^E}\right) + \frac{G^E c^{EE} r^E + G^E I^{ext} - \mu^E}{c^{EI}}
\end{aligned}
\tag{25}
$$

where log denotes the natural logarithm and exp the exponential function.

Similarly, we can derive the nullcline of r^I by equating $\frac{dr^I}{dt}$ to 0, yielding:

$$
\begin{aligned}
0 &= \frac{dr^I}{dt} \\
0 &= -r^I + F^I\left(c^{IE} r^E\right) \\
r^I &= \frac{1}{1 + exp(-(c^{IE} r^E - \mu^I)/\sigma^I)}
\end{aligned}
\tag{26}
$$
