# Supplementary material for: Excitatory-inhibitory homeostasis and bifurcation control in the Wilson-Cowan model of cortical dynamics
Source: PLoS Comput Biol. 2025 Jan 6;21(1):e1012723. doi: 10.1371/journal.pcbi.1012723 (PMC11737862; doi:10.1371/journal.pcbi.1012723)
Supplement: S3 Appendix — (PDF) [file pcbi.1012723.s011.pdf]

S3 Appendix Homeostasis of Excitatory Synapses Unto Inhibitory Neurons

Although the evidence is scarce, some studies indicate that fast-spiking interneurons may also scale the strength of their excitatory synapses [43] and that this process depends mainly on pre-synaptic activity, with the function of maintaining PY firing rates [60]. This type of homeostasis can be implemented by scaling the parameter c^{IE}, which quantifies the strength of the excitatory inputs to the inhibitory neural mass. By reordering Eq (5), we obtain:

$$c^{IE} = \frac{1}{r^E}\left(\mu^I - \sigma^I log\left(\frac{1}{\frac{\sigma^E}{c^{EI}} log\left(\frac{1-r^E}{r^E}\right) + \frac{G^E c^{EE} r^E + G^E I^{ext} - \mu^E}{c^{EI}}} - 1\right)\right) \tag{27}$$

The most notable difference of c^{IE} homeostasis relative to the two other types of synaptic scaling is the lack of robustness to higher levels of external input (Figure S3 Fig). More specifically, if $I^{ext} \gtrsim 2.65$, the system is not able to maintain any desired fixed point r^E_{fixed} through homeostasis of excitatory synapses unto the inhibitory population (see blank space in Figure S3 Fig). This finding can be trivially explained given the dynamics of our model. For this type of homeostasis, when excitatory activity is too high, the model increases the excitation of inhibitory populations so that they inhibit the excitatory population more. However, because of the sigmoid non-linearity of the model, there is a cap on the activity of the inhibitory population at $r^I = 1$. Therefore, regardless of how much the inhibitory population is excited, the maximum inhibitory input it can provide to the excitatory neural mass is equal to $c^{EI} \times 1$. For this reason, after the point where homeostasis requires the saturation of the inhibitory population to maintain r^E_{fixed}, the system can no longer respond to increases in I^{ext} and, thus, it is not able to maintain homeostasis of excitatory activity. For this reason, we do not consider this mode of homeostasis in our further exploration of E-I balance in the WC model.
