# Supplementary material for: Excitatory-inhibitory homeostasis and bifurcation control in the Wilson-Cowan model of cortical dynamics
Source: PLoS Comput Biol. 2025 Jan 6;21(1):e1012723. doi: 10.1371/journal.pcbi.1012723 (PMC11737862; doi:10.1371/journal.pcbi.1012723)
Supplement: S4 Appendix — (PDF) [file pcbi.1012723.s012.pdf]

S4 Appendix Computing steady-state parameters in systems with multiple modes of homeostasis

To derive an analytical method to compute the steady-state parameters of models under multiple modes of homeostasis we start by defining equations describing the dynamics of homeostasis. Starting with plasticity of G^E and c^{EI}, we can write their dynamics as:

$$\begin{aligned} \tau_{homeo}\frac{dG^E}{dt} &= -(r^E - \rho) \\ \tau_{homeo}\frac{dc^{EI}}{dt} &= (r^E - \rho) \end{aligned} \tag{28}$$

Shortly, if the firing rate of the excitatory neural mass r^E is larger than the target firing rate ρ, G^E will decrease and c^{EI} will increase, bringing the firing rate down due to reduced excitation and enhanced inhibition. While this implementation of c^{EI} homeostasis differs from the common approach in large-scale models [36, 37, 39, 100], in S5 Appendix we argue that it is more sensible for this version of the Wilson-Cowan model. Given these equations, one can write the value of G^E at time t as follows:

$$\begin{aligned} G^E(t) &= G^E_0 + \int_0^t -\frac{1}{\tau_{homeo}}(r^E - \rho)dt \\ G^E(t) &= G^E_0 - \frac{1}{\tau_{homeo}}\int_0^t (r^E - \rho)dt \end{aligned} \tag{29}$$

where G^E_0 is the initial value of G^E at $t = 0$. Similary, we can write $c^{EI}(t)$ as:

$$c^{EI}(t) = c^{EI}_0 + \frac{1}{\tau_{homeo}}\int_0^t (r^E - \rho)dt \tag{30}$$

Considering that the term inside the integral is the same in Eqs 29 and 30, we can write:

$$G^E_0 - G^E(t) = c^{EI}(t) - c^{EI}_0 \tag{31}$$

Therefore, in simpler terms, the magnitude of variation in both parameters between any two points in time is the same, provided that the timescales of homeostatic plasticity (τ_{homeo}) are also the same.

Similarly, for modes of plasticity involving the modulation of intrinsic excitability, and considering that, if firing rates are higher than ρ, the firing threshold μ^E should increase, we can write the dynamics of μ^E as:

$$\tau_{homeo}\frac{d\mu^E}{dt} = (r^E - \rho) \tag{32}$$

leading to:

$$\mu^E(t) = \mu^E_0 + \frac{1}{\tau_{homeo}}\int_0^t (r^E - \rho)dt \tag{33}$$

Then, we can write the following equality, relating μ^E to G^E and c^{EI}:

$$G_0^E - G^E(t) = c^{EI}(t) - c_0^{EI} = \mu^E(t) - \mu_0^E \tag{34}$$

However, up until now, we have assumed that all forms of homeostasis operate in the same timescales. That said, it is still possible to solve the equations when the timescales are different. Let us assume that the timescales of c^{EI} and μ^E can be related to the timescale of G^E (τ_{homeo}) through the ratios R_1 and R_2, respectively. In that case, we write:

$$\begin{aligned} G^E(t) &= G_0^E - \frac{1}{\tau_{homeo}} \int_0^t (r^E - \rho)dt \\ c^{EI}(t) &= c_0^{EI} + \frac{1}{R_1 \tau_{homeo}} \int_0^t (r^E - \rho)dt \\ \mu^E(t) &= \mu_0^E + \frac{1}{R_2 \tau_{homeo}} \int_0^t (r^E - \rho)dt \end{aligned} \tag{35}$$

which can be rewritten as:

$$\begin{aligned} G_0^E - G^E(t) &= \frac{1}{\tau_{homeo}} \int_0^t (r^E - \rho)dt \\ R_1(c^{EI}(t) - c_0^{EI}) &= \frac{1}{\tau_{homeo}} \int_0^t (r^E - \rho)dt \\ R_2(\mu^E(t) - \mu_0^E) &= \frac{1}{\tau_{homeo}} \int_0^t (r^E - \rho)dt \end{aligned} \tag{36}$$

Therefore, we can re-write the previous equality (Eq. 34) as:

$$G_0^E - G^E(t) = R_1(c^{EI}(t) - c_0^{EI}) = R_2(\mu^E(t) - \mu_0^E) \tag{37}$$

This can be then employed to derive the new equations determining the steady-state values of the model parameters under multiple modes of homeostasis considering that the timescales of each mode can be different. That considered, for the plasticity of G^E and c^{EI}, we have:

$$\begin{aligned} c^{EI} &= \frac{\sigma^E log\left(\frac{1-r^E}{r^E}\right) + (G_0^E + R_1 c_0^{EI})(c^{EE} r^E + I^{ext}) - \mu^E}{F^I(c^{IE} r^E) + R_1 c^{EE} r^E + R_1 I^{ext}} \\ G^E &= G_0^E + R_1(c^{EI}(t) - c_0^{EI}) \end{aligned} \tag{38}$$

Similarly, for plasticity of G^E, c^{EI} and μ^E, we obtain:

$$\begin{aligned} \mu^E &= \frac{\sigma^E log\left(\frac{1-r^E}{r^E}\right) - (c_0^{EI} - \frac{R_2}{R_1}\mu_0^E) F^I(c^{IE} r^E) + (G_0^E + R_2 \mu_0^E)(c^{EE} r^E + I^{ext})}{1 + \frac{R_2}{R_1} F^I(c^{IE} r^E) + R_2 c^{EE} r^E + R_2 I^{ext}} \\ G^E &= G_0^E + R_2(\mu^E(t) - \mu_0^E) \\ c^{EI} &= c_0^{EI} + \frac{R_2}{R_1}(\mu^E(t) - \mu_0^E) \end{aligned} \tag{39}$$

Finally, for plasticity of G^E, c^{EI}, μ^E and σ^E, we reach the following expression:

$$
\begin{aligned}
\mu^E &= \frac{(c_0^{EI} - \frac{R_2}{R_1}\mu_0^E)F^I(c^{IE}r^E) - (G_0^E + R_2\mu_0^E)(c^{EE}r^E + I^{ext})}{Klog\left(\frac{1-rE}{rE}\right) - 1 - \frac{R_2}{R_1}F^I(c^{IE}r^E) - R_2c^{EE}r^E - R_2I^{ext}} \\
G^E &= G_0^E + R_2(\mu^E(t) - \mu_0^E) \\
c^{EI} &= c_0^{EI} + \frac{R_2}{R_1}(\mu^E(t) - \mu_0^E)
\end{aligned} \tag{40}
$$

where $K = \frac{\sigma_0^E}{\mu_0^E}$. In S4 Fig, we demonstrate that our analytical method can accurately predict the steady-state values of model parameters with different initial values and time constants.
