# Supplementary material for: Excitatory-inhibitory homeostasis and bifurcation control in the Wilson-Cowan model of cortical dynamics
Source: PLoS Comput Biol. 2025 Jan 6;21(1):e1012723. doi: 10.1371/journal.pcbi.1012723 (PMC11737862; doi:10.1371/journal.pcbi.1012723)
Supplement: S5 Appendix — (PDF) [file pcbi.1012723.s013.pdf]

S5 Appendix Dynamics of c^{EI} Homeostasis

The most common implementation of homeostatic plasticity of inhibition relies on the fact that this plasticity can be disabled by abolishing firing in both the post-synaptic excitatory and pre-synaptic inhibitory neurons [17]. For this reason, [14] implemented this mode of homeostasis for feedforward inhibitory synapses in the context of networks of rate-based neurons with the following formulation:

$$\tau_{homeo} \frac{dc^{EI}}{dt} = r^I (r^E - \rho) \tag{41}$$

where ρ represents the target average firing rate. This implementation has been later translated into the context of large-scale modeling, particularly in approaches relying on the Wilson-Cowan model or similar neural-mass models [36, 37, 39–41, 100]. However, here we argue that this is may not the most appropriate approach for the current implementation of the Wilson-Cowan model.

Let us start by considering that the time constant of homeostatic plasticity is normally orders of magnitude slower than the timescale of population activity, ensuring the separation of timescales. Therefore, for the analysis of homeostasis, it can be considered that the dynamics of Equation 41 dominate the system, while r^E and r^I quickly reach their respective steady-states, where $r^I = F^I(c^{IE} r^E)$. Therefore, we can write Equation 41 as:

$$\tau_{homeo} \frac{dc^{EI}}{dt} = F^I(c^{IE} r^E)(r^E - \rho) \tag{42}$$

Now, let us consider that we have two different initial systems for which the steady-state firing rate $r_0^E = \rho - d$ and $r_1^E = \rho + d$, with $d > 0$. Therefore, we have:

$$\begin{aligned} \tau_{homeo} \frac{dc_0^{EI}}{dt} &= F^I(c^{IE}(\rho - d))(-d) \\ \tau_{homeo} \frac{dc_1^{EI}}{dt} &= F^I(c^{IE}(\rho + d))(d) \end{aligned} \tag{43}$$

Given that F^I is sigmoid and, thus, a monotonously increasing function, we have that:

$$F^I(c^{IE}(\rho - d)) < F^I(c^{IE}(\rho + d)) \tag{44}$$

which means that:

$$\begin{aligned} \left| F^I(c^{IE}(\rho - d))(-d) \right| &< \left| F^I(c^{IE}(\rho + d))(d) \right| \\ \left| \frac{dc_0^{EI}}{dt} \right| &< \left| \frac{dc_1^{EI}}{dt} \right| \end{aligned} \tag{45}$$

Therefore, in two systems where the firing rate is equally distant from ρ, the one where the firing rate is lower than ρ will necessarily converge more slowly toward the target than the one where r^E is above ρ. For this reason, under this implementation of homeostasis, balanced WC nodes recover more slowly from decreases in firing rates than from increases. However, there is no empirical evidence for this behavior, with results suggesting that homeostasis of inhibition operates in similar timescales regardless of the direction of perturbation [17, 20, 21, 59, 101].

We illustrate the influence of the r^I term on the derivative of c^{EI} in S5 Fig and compare it with the case where $\tau_{homeo}\frac{dc^{EI}}{dt} = r^E - \rho$. Here, the aforementioned behavior can be observed more clearly, since the magnitude of the derivative of c^{EI} is considerably lower when $r^E < \rho = 0.1$. In addition, comparison with $r^E - \rho$ allows for the visualization of a further issue with this implementation. Since the sigmoid non-linearity is bound between 0 and 1, $r^I(r^E - \rho) < (r^E - \rho)$, this form of plasticity will always be slower than modes that are only dependent on the difference between r^E and ρ, such as synaptic scaling of excitation [17, 19, 43], even when considering equal timescales. This effect is more pronounced for values of r^E lower than ρ, since r^I approaches 0, further slowing down the homeostasis of c^{EI}.

For these reasons, we consider that, in the current implementation of the WC model, the most reasonable expression for c^{EI} homeostasis is:

$$\tau_{homeo}\frac{dc^{EI}}{dt} = r^E - \rho \tag{46}$$

as opposed to the most common implementation [36, 37, 39–41, 100]. Furthermore, we argue that it may still be in line with literature. More specifically, [17] states that *"in contrast to synaptic scaling of excitatory synapses, homeostatic regulation of inhibition is a non-cell-autonomous process that either requires changes in both pre- and postsynaptic activity simultaneously or is triggered by global changes in network activity."*. In our case, since we are working with mean-field models, which abstract the aggregate network activity into a single variable r^E or r^I, it can be considered that r^E is sufficient to track network activity. Not only that, but, given the separation in timescales between neural dynamics and homeostasis, r^I can be understood as $F^I(c^{IE}r^E)$, which means that perturbing r^E will lead to a perturbation of r^I in the same direction, scaled by F^I. For these reasons, we consider that Equation 46 represents a reasonable implementation of homeostasis of inhibition in the Wilson-Cowan model, and possibly other mean-field approaches, particularly when activity is constrained by a sigmoid non-linearity.
