# Supplementary material for: Excitatory-inhibitory homeostasis and bifurcation control in the Wilson-Cowan model of cortical dynamics
Source: PLoS Comput Biol. 2025 Jan 6;21(1):e1012723. doi: 10.1371/journal.pcbi.1012723 (PMC11737862; doi:10.1371/journal.pcbi.1012723)
Supplement: S6 Appendix — (PDF) [file pcbi.1012723.s014.pdf]

S6 Appendix Derivation of Jacobian Matrix for the Wilson-Cowan Model

Let us consider that the equations of the Wilson-Cowan system can be written as:

$$\begin{aligned} \frac{dr^E}{dt} &= f(r^E, r^I) \\ \frac{dr^I}{dt} &= g(r^E, r^I) \end{aligned} \tag{47}$$

where:

$$\begin{aligned} f(r^E, r^I) &= \frac{1}{\tau^E}\left(-r^E + F^E\left(G^E c^{EE} r^E - c^{EI} r^I + G^E I^{ext}\right)\right) \\ g(r^E, r^I) &= \frac{1}{\tau^I}\left(-r^I + F^I\left(c^{IE} r^E\right)\right) \end{aligned} \tag{48}$$

Then, the first-order part of the Taylor series of the Wilson-Cowan around a given point $a = \begin{pmatrix} r^E_{fixed} \\ r^I_{fixed} \end{pmatrix}$ can be written as:

$$\begin{bmatrix} \frac{dr^E}{dt} \\ \frac{dr^I}{dt} \end{bmatrix} = \cancelto{0}{\begin{bmatrix} f(a) \\ g(a) \end{bmatrix}} + \begin{bmatrix} \frac{df(a)}{dr^E} & \frac{df(a)}{dr^I} \\ \frac{dg(a)}{dr^E} & \frac{dg(a)}{dr^I} \end{bmatrix} (x - a) \tag{49}$$

Given that a is, by definition, a fixed point of the system $f(a) = g(a) = 0$. Therefore, we have a linear approximation of the system around a, from which we obtain the Jacobian matrix (J) containing the partial derivatives of $f(x)$ and $g(x)$. The partial derivatives of each function can be expressed as a function of the model parameters as follows.

$$\frac{df}{dr^E} = -\frac{1}{\tau^E} + \frac{d}{dr^E}\left(F^E\left(G^E c^{EE} r^E - c^{EI} r^I + G^E I^{ext}\right)\right) \tag{50}$$

Furthermore, since the derivative of the canonical sigmoid function $F(x) = \frac{1}{1+e^{-x}}$ is $F(x)(1 - F(x))$, we can use the chain rule to derive the second part of $\frac{df}{dr^E}$ as follows, where $x = G^E c^{EE} r^E - c^{EI} r^I + G^E I^{ext}$:

$$\begin{aligned} \frac{df}{dr^E} &= -\frac{1}{\tau^E} + \frac{d}{dr^E}\left(\frac{x - \mu^E}{\sigma^E}\right) F^E(x)\left(1 - F^E(x)\right) \\ \frac{df}{dr^E} &= -\frac{1}{\tau^E} + \frac{G^E c^{EE}}{\sigma^E} F^E(x)\left(1 - F^E(x)\right) \end{aligned} \tag{51}$$

Similarly, we can derive the other components of the Jacobian matrix as:

$$\begin{aligned} \frac{df}{dr^I} &= \frac{d}{dr^I}\left(\frac{x - \mu^E}{\sigma^E}\right) F^E(x)\left(1 - F^E(x)\right) \\ \frac{df}{dr^I} &= -\frac{c^{EI}}{\sigma^E} F^E(x)\left(1 - F^E(x)\right) \end{aligned} \tag{52}$$

$$\begin{aligned} \frac{dg}{dr^E} &= \frac{d}{dr^E}\left(\frac{c^{IE} r^E - \mu^I}{\sigma^I}\right) F^I(c^{IE} r^E)\left(1 - F^I(c^{IE} r^E)\right) \\ \frac{dg}{dr^E} &= \frac{c^{IE}}{\sigma^I} F^I(c^{IE} r^E)\left(1 - F^I(x)\right) \end{aligned} \tag{53}$$

$$\begin{aligned} \frac{dg}{dr^I} &= -\frac{1}{\tau^I} + \cancelto{0}{\frac{d}{dr^I}\left(F^I(c^{IE} r^E)\right)} \\ \frac{dg}{dr^E} &= -\frac{1}{\tau^I} \end{aligned} \tag{54}$$
