# Supplementary material for: Excitatory-inhibitory homeostasis and bifurcation control in the Wilson-Cowan model of cortical dynamics
Source: PLoS Comput Biol. 2025 Jan 6;21(1):e1012723. doi: 10.1371/journal.pcbi.1012723 (PMC11737862; doi:10.1371/journal.pcbi.1012723)
Supplement: S7 Appendix — (PDF) [file pcbi.1012723.s015.pdf]

S7 Appendix Analytical Derivation of Oscillation Frequency Around a Fixed Point

In a given fixed point of any 2-dimensional dynamical system, if the eigenvalues of the Jacobian have an imaginary component (i.e. $Tr(J)^2 - 4Det(J) < 0$), the system will have a rhythmic behavior and the frequency of oscillation around the fixed point can be estimated from J. If we consider $-4\omega^2 = Tr(J)^2 - 4Det(J)$, and that $\omega > 0$ then the solution to the eigenvalue problem of the system yields:

$$\lambda^{\pm} = \frac{Tr(J) \pm \sqrt{-4\omega^2}}{2} = \frac{Tr(J) \pm 2i\omega}{2} \tag{55}$$

which corresponds to the following solution of the system, where $v^{\pm}$ represents the eigenvectors of the Jacobian:

$$x(t) = v^{\pm} e^{\frac{(Tr(J) \pm 2i\omega)t}{2}} = v^{\pm} e^{\frac{Tr(J)t}{2}} e^{i\omega t} \tag{56}$$

Therefore, given that the $e^{i\omega t}$ term represents a sinusoidal wave in time, we can estimate the frequency of oscillation around the fixed point from the Jacobian matrix, given that $\omega = 2\pi f$, as:

$$f = \frac{\omega}{2\pi} = \frac{\sqrt{4Det(J) - Tr(J)^2}}{4\pi} \tag{57}$$

This approximation, however, is only valid around the fixed point. For example, in systems with a repelling fixed point, where dynamics settle on a limit cycle trajectory, the frequency of oscillation will decrease as the system orbits away from the fixed point (S6 Fig).
