# Supplementary material for: Excitatory-inhibitory homeostasis and bifurcation control in the Wilson-Cowan model of cortical dynamics
Source: PLoS Comput Biol. 2025 Jan 6;21(1):e1012723. doi: 10.1371/journal.pcbi.1012723 (PMC11737862; doi:10.1371/journal.pcbi.1012723)
Supplement: Attachment — Submitted filename: Reviewer_Response.pdf [file pcbi.1012723.s017.pdf]

Dear Editors,

We thank you for the opportunity to resubmit a revised version of the manuscript for publication in the PLOS Computational Biology journal. We appreciate the effort and time that you and the reviewers have taken to provide feedback on our manuscript. That said, we have been able to update it to account for most of the suggested revisions, which we believe have improved the document. Thus, we provide two versions of the revised manuscript: with and without tracked changes. In addition, we include below a point-by-point response to the reviewer's concerns together with the respective amendments to the manuscript, written in ***blue***. Importantly, in all the responses below, the lines indicating the amended parts of the revised manuscript refer to the version **without tracked changes**. Finally, we have also edited the ordering of the supplementary figures and text, to better reflect their order of appearance in the text.

Comments from Reviewer 1

This study scrutinizes the effects of various modes of E-I homeostasis on cortical dynamics by using well-established concepts of dynamical systems theory. The study design and findings are consistent. The presented results support the claims. The manuscript is well written. There are a few questions and suggestions that the authors are encouraged to take into account:

Major Issues

Q1.1 - *Page 3, line 83, and Eq. (1): The term G^E in Eq. (1) was introduced to o control the gain of all excitatory inputs. However, in table 1, it was assigned a value equal to 1, but later in line 155, its value was changed and it was linked to the stabilization of excitatory firing rate corresponding to a fixed point under the external current. Was is the implication of G^E = 1?*

> **R1.1 –** In this manuscript, we update the canonical Wilson-Cowan equations by including the term G^E, which allows for scaling all excitatory synapses (recurrent excitation and external excitatory inputs). This was done in order to be able to simulate the effects of excitatory synaptic scaling by adjusting G^E to maintain a target firing rate, following the principles of synaptic scaling. A model with G^E = 1 would be effectively equal to the canonical Wilson-Cowan model without this gain term and, in Table 1, we present this value as the default for G^E, as we do with other parameters that can also be modulated by other modes of homeostasis (e.g. c^EI). Furthermore, we point out that, for the isolated homeostasis of G^E, the homeostatic value is independent of the initial value of the parameter. While this is not the case when multiple homeostatic mechanisms interact, we believe that the exploration of different initial values is beyond the scope of this paper and would not substantially add to the mechanistic understanding of how E-I homeostasis shapes circuit dynamics in cortical networks. Nonetheless, we have

added the following text to the discussion to comment on this limitation of our study:

L570-578: On the other hand, the homeostatic set-point of circuits with multiple homeostatic mechanisms is also dependent on the initial value of each parameter (S4 Appendix). This raises the question of how much these values shape the steady-state dynamics of the system and what is the range of initial conditions tolerated by the circuit without detriment to its behavior. Therefore, we suggest that future computational studies should focus not only on this question, but also on fitting the default parameters of the model with population recordings from cortical slices, where the external input can be easily controlled. That said, we believe that our results serve to provide the mathematical framework to study these questions in the future.

Q1.2 - *Page 5, Fig. 1b: It would be nice to extract the values of r^E in Fig. 1b and indicate them either in the figure or in the caption of the figure.*

R1.2 – We thank the reviewer for their comment. We have thus added the values for r^E in the fixed points to Figure 1b to improve clarity.

Q1.3 - *This study focuses primarily on how cortical dynamics are shaped by the diverse mechanisms of E-I homeostasis. But, it would be worthy to also discuss the relation of homeostatic plasticity (that stabilizes network activity) to Hebbian plasticity (structural as well functional) that shapes network connectivity (see, for example, Gallinaro & Rotter, 2018, Scientific Reports). It would be crucial to acknowledge that various plasticity mechanisms in different forms work in concert to shape cortical dynamics.*

R1.3 – We thank the reviewer for pointing out this issue. Indeed, several neural mechanisms operate at different scales and shape the dynamics of cortical networks. For example, in line 691 of the original manuscript, we discuss how the slow homeostasis of E-I balance may interact with faster mechanisms for the modulation of E-I interactions. However, we agree that the discussion of other plasticity mechanisms is lacking and, therefore, we have added the following excerpt to the discussion:

L815-821: On another note, it should also be pointed out that homeostatic plasticity is known to interact with other learning mechanisms, such as Hebbian plasticity, contributing to the stabilization of both firing-rates and memory engrams [14, 85]. Nonetheless, other studies highlight the relevance of homeostatic plasticity, suggesting that Hebbian-like learning can emerge in networks with only structural mechanisms of firing-rate homeostasis [86, 87], and that homeostatic plasticity explains the organization of connectivity in motor areas better than Hebbian mechanisms [88].

Q1.4 - *Page 25, line 634, Effect of time scale of the inhibitory synapses on the emergence of oscillatory dynamics have been focus of several papers that could be refered. See e. g., Khanjanianpak et al, iScience 2024; Roohi and Valizadeh, Neural computation 2023.*

R1.4 – We thank the reviewer for the suggestions, which we have added to the discussion of the manuscript with a short contextualization.
L677-686: The study of the effects of inhibitory synaptic timescales in cortical oscillatory dynamics is not new. Previous studies demonstrate that gamma oscillations can emerge in E-I loops if inhibition has a slower decay than excitation [74], favoring oscillations in the low gamma range [75]. Furthermore, longer inhibitory time constants also favor the emergence of complex oscillatory dynamics, with noise-driven switching between regimes of intermittent and sustained rhythms [76]. However, although it has been suggested that the interaction between synaptic time constants and E-I balance is pivotal in determining cortical rhythms [75], we are the first, to our knowledge, to explore this interaction in systems where the balance is actively maintained through E-I homeostasis. In such circuits, our results suggest that…

Minor Issues

Q1.5 - Could the authors please re-adjust the figures in the manuscript to be positioned either at the top or bottom of the page?

R1.5 – The manuscript was produced using the PLOS CB latex template and would be edited according to the journal's template in its published form. Therefore, we decided to maintain the draft as it is for the moment.

Q1.6 - Caption of Fig. 2: A period is missing after "… in the Wilson-Cowan Model".

R1.6 – This mistake was fixed in the revised version.

Q1.7 - *Page 7, lines 157-158: "Another common form of E-I homeostasis is the synaptic scaling of fast-spiking 157 inhibitory synapses onto pyramidal neurons [12, 17, 21] (Fig 2).". Please clearly indicated which panel in Fig. 2.*

R1.7 – This mistake was fixed in the revised version of the manuscript.
L168: (Fig 2, middle panel)

Q1.8 - *Page 8, line 196: "Here, we present a heuristic process or method or something like that …" instead of "Here, we present a heuristic …".*

R1.8 – We have modified the text in the revised manuscript

Q1.9 - *Caption of Fig. 3: There is an extra period just after I^ext = 2.*

R1.8 – This mistake was fixed in the revised version of the manuscript.

Comments from Reviewer 2

This paper investigates the role of excitatory-inhibitory (E-I) homeostasis in shaping the dynamics of cortical networks, particularly focusing on how different homeostatic mechanisms influence oscillations and the system's proximity to a bifurcation point. The authors employ the Wilson-Cowan (WC) model, a classic model of cortical dynamics, and extend it to incorporate various modes of E-I homeostasis. They derive analytical solutions for the fixed points of the system under different homeostatic mechanisms and use bifurcation analysis to characterize the system's behaviour.
The key findings are:

- ***E-I homeostasis can control the system's proximity to a bifurcation point**, influencing whether the system exhibits damped oscillations, sustained oscillations, or stable fixed points.*
- ***Different modes of homeostasis have distinct effects on the bifurcation point**. Some modes shift the bifurcation towards higher firing rates with increased external input, while others shift it towards lower firing rates or keep it independent of input.*
- ***Multiple modes of homeostasis acting in conjunction can lead to more robust regulation of network dynamics** and keep oscillation frequencies within a physiologically plausible range.*
- ***The time constant of inhibition also influences the bifurcation point**, with slower inhibition making sustained oscillations more likely.*

*The most interesting claims, in my view, are those related to the **heterogeneity of bifurcation points across cortical areas** and the **functional benefits of employing multiple homeostatic mechanisms**. These claims provide potential explanations for observed phenomena in the cortex and offer insights into the design principles of cortical networks.*
The results presented in this paper are intriguing and potentially significant. However, there are a few points that require further clarification and investigation to strengthen the conclusions.

Major Issues:

Q2.1 - *Generality of the findings: The study focuses on the WC model, which is a simplified representation of cortical dynamics. While the WC model captures essential features of E-I interactions, it would be important to discuss the extent to which the findings can be generalized to more complex and biologically realistic models of cortical networks.*

> **R2.1 –** We thank the reviewer for this point. A more precise exploration of the generality of our findings would require many simulations using a network model of, for example, integrate-and-fire neurons, which would take a considerable amount of time. Nonetheless, we believe that there are examples in the literature of studies implementing similar mechanisms of homeostasis in models with detailed neuronal dynamics, showing that E-I homeostasis can shape circuit dynamics in a way similar to our results (e.g. bifurcation control). Therefore, we believe that our results are most likely generalizable. However, we agree that this

should be subject to further investigation and have updated the discussion to account for this limitation of our approach.

L776-799: In this work, we have focused on mean-field circuit dynamics, demonstrating the effects of multiple homeostatic mechanisms for the mean activity of populations of thousands of excitatory and inhibitory neurons. The main reason for this choice is the potential of neural-mass models to be used in the study of the effects of E-I homeostasis in the large-scale dynamics of cortical networks [36, 39–41]. However, such studies have focused mainly on the plasticity of inhibitory synapses, neglecting the potential impact of further homeostatic mechanisms. For that reason, we developed the current framework to be implemented in large-scale models of the human cortex, investigating the specific contributions of different homeostatic mechanisms to large-scale cortical dynamics. That said, it should be investigated if our findings extend to more detailed models accounting for neuronal spiking and more complex synaptic dynamics. In this context, we point out that the Wilson-Cowan model has been demonstrated to capture a wide range of aspects of cortical dynamics [45], suggesting that it is an appropriate framework for our study. However, we also point out that homeostatic plasticity has been similarly implemented in more detailed spiking and firing-rate models of the cortical neurons [14, 26, 89–91], showing that it can not only maintain the mean firing rate of neurons but also have effects on circuit dynamics, such as the distance-to-bifurcation [26], in line with our results. Therefore, we believe that our conclusions might still apply to more detailed models of cortical networks. Nonetheless, future investigation is warranted, focusing on mechanisms of homeostasis that have not yet been implemented in spiking models (e.g. the co-modulation of μE and σE) and in performing a direct comparison between the effects of E-I homeostasis in models that reproduce cortical dynamics at different scales, from the spiking activity of single neurons to whole-brain dynamics.

Q2.2 - *Novelty of the multiple homeostasis approach: The authors claim that their approach of considering multiple homeostatic mechanisms acting in conjunction is novel. While this is a valuable contribution, it would be beneficial to provide a more thorough discussion of how this approach differs from previous studies that have also considered multiple homeostatic mechanisms (e.g., Cannon, Jonathan, and Paul Miller. "Synaptic and intrinsic homeostasis cooperate to optimize single neuron response properties and tune integrator circuits." Journal of neurophysiology 116.5 (2016): 2004-2022).*

R2.2 – We thank the reviewer for pointing out this issue. Indeed, we agree that the discussion on previous computational studies exploring the interaction between multiple homeostatic mechanisms was lacking. We have, therefore, updated the discussion section to comment on how our approach differs from previous studies. In addition, we have also extended the discussion on homeostatic plasticity in inhibitory neurons, which we have not studied in detail, highlighting relevant studies and suggesting further study

L728-775: On another note, it should be stated that this study is not the first to investigate the benefits of employing multiple homeostatic mechanisms on cortical dynamics, although previous computational studies have focused mainly

on networks of spiking neurons [26, 89, 90]. Combining the synaptic scaling of excitatory synapses and the plasticity of firing thresholds has been shown to optimize mutual information between neuronal inputs and outputs better than individual homeostatic mechanisms, contributing to the adaptation to perturbations in stimulus statistics [89]. Whether these benefits are present or not at the level of mean population activity remains to be investigated in future studies using our methodological framework. Furthermore, studies in spiking networks demonstrate that each mechanism has a specific contribution to circuit dynamics [90]. More specifically, synaptic scaling is relevant for the recovery of correlations between spontaneous activity. However, our methodology does not allow for the investigation of correlation structures, since population activity is abstracted in a single variable (rE or rI). Conversely, the plasticity of firing thresholds was found to contribute more strongly to the maintenance of mean firing rates [90]. Here, we argue that this finding is in line with our results suggesting the plasticity of μ^E can robustly maintain mean firing rates, regardless of input levels, without affecting circuit dynamics (see Fig 4c). That said, we stress that most studies show that the plasticity of intrinsic excitability is rather achieved through the synergistic modulation of both firing thresholds and slopes [23–25]. Therefore, we propose that a mechanism that modulates both μ^E and σ^E is more appropriate, although its functional advantages are not yet clear. In addition, it should be pointed out that further modeling results also suggest that scaling of excitation and inhibition in pyramidal neurons can also regulate firing rates effectively, although the plasticity of intrinsic excitability was not accounted for [26].

In this study, although we derived the mathematical solution for cIE homeostasis, we did not delve deeply into the study of firing-rage homeostasis in inhibitory interneurons. However, research suggests that firing-rate homeostasis in fast-spiking interneurons not only occurs, but is faster than excitatory homeostasis, providing a mechanism for fast regulation of network dynamics following perturbations [42, 48]. That said, the precise mechanisms of inhibitory firing-rate homeostasis are unknown. While some results point out that the excitatory synapses in PV neurons are also scaled in response to perturbations in PY firing rates [43, 60], this mechanism may only come into play when activity is higher than the target [43]. Conversely, our analysis suggests that, at least for the WC model, it is not a sufficiently robust mode of homeostasis precisely when perturbations (i.e. higher Iext) lead to higher firing rates. This is because homeostasis of c^IE acts through the disynaptic modulation of E firing rates by increasing the excitation of the inhibitory population, which provides feedback inhibition. Since inhibitory activity has an upper limit due to the sigmoid non-linearity of the model, this mode of homeostasis breaks down under higher levels of external input (see S4 Appendix). However, it is possible that this mode of homeostasis, in combination with other forms of plasticity, can contribute to the maintenance of circuit dynamics. For example, the plasticity of inhibitory-to-inhibitory synapses has been shown to contribute to the maintenance of network criticality after sensory deprivation in a spiking model of the visual cortex with multiple homeostatic mechanisms [26]. Therefore, given that our model can be adapted to include self-inhibition (S1 Appendix), we suggest that our approach

can be used to study the modulation of both c^IE and c^II for inhibitory firing-rate homeostasis and its effects on circuit dynamics.

Q2.3 - *Additional benefits: The functional benefits of employing multiple homeostatic mechanisms could further be highlighted by, for example: Exploring the effects of different homeostatic timescales on network dynamics and stability; quantifying the robustness of network dynamics to perturbations under different combinations of homeostatic mechanisms to demonstrate the benefits of multiple homeostasis.*

R2.3 – We agree with the reviewer that some of the benefits of multiple homeostatic mechanisms could be further highlighted. An example that is already mentioned in the manuscript is explaining the correlation between inhibition and the frequency of gamma oscillations, while maintain such frequencies within realistic bounds (Kujala et al 2015, 2024), which can only occurs when accounting for the homeostasis of excitation and inhibition at least. Therefore, have decided to change figure S4 from the supplementary material to the main text as Figure 6b, adding the following text to the Results section to provide context for the figure and highlight the benefits of employing multiple homeostatic mechanisms:

L455-475: On another note, studies have demonstrated that the frequency of gamma oscillations in different areas of the human cortex correlates significantly with the density of inhibitory receptors [63, 64]. Importantly, although this relationship is found, the frequency of physiological low-gamma oscillations remains roughly bounded between 30 and 100 Hz. To investigate if this interaction is also found in our neural-mass model, we plot the peak frequency of gamma oscillations against the inhibitory coupling cEI in all models involving the homeostasis of inhibition (Fig 6b). Our results demonstrate that the empirical relationship between inhibition and gamma frequencies is a general feature of our model, regardless of the combination of homeostatic parameters or the target firing rate. However, as previously pointed out, in models with the plasticity of inhibition only (Fig 6b), the frequency goes beyond the range reported in [63, 64]. Conversely, when relying on multiple homeostatic mechanisms, cortical circuits maintain gamma rhythms within physiological bounds (Fig 6b), further showcasing the circuit-level benefits of combining multiple mechanisms for firing-rate homeostasis. All in all, we demonstrate that, beyond the maintenance of target firing rates, different modes of homeostasis can have profound impacts on node dynamics such as the frequency of oscillations. Importantly, this result has implications for the oscillatory activity in cortical areas with different densities of excitatory and inhibitory receptors, which might arise due to the effect of E-I homeostasis. Therefore, we stress that this effect should be considered when interpreting results showing a relationship between gamma oscillation frequency with the density of inhibitory receptors in the cortex.

Regarding the effect of changing the homeostatic timescales on circuit dynamics, we would first point out that the separation of timescales between homeostasis and neuronal dynamics must be ensured, given the empirically attested timescales of E-I homeostasis (consult Turrigiano 2011 and Wen and Turrigiano 2024 for a review of the topic). Given such separation of time scales, in circuits with single mechanisms of homeostasis, the system will always settle in the same fixed point regardless of the timescale, since the dynamics of the system are dominated by homeostatic dynamics (e.g. $\tau_{homeo}\frac{dG^E}{dt} = \rho - r^E$), where the fixed point does not depend on τ_{homeo}. Conversely, in systems with multiple mechanisms of homeostasis, the homeostatic fixed point is indeed determined by the relative timescales of the different mechanisms. In this case, the fastest mechanisms will dominate the dynamics. We touch on this issue in S3 Appendix, when deriving the analytical solution for models with multiple homeostatic mechanisms. For example, in S3 Appendix, Figure 12, we show that, in general, if homeostasis of a given parameter is faster, the system will mostly rely on this parameter to maintain mean firing rates. However, given that previous studies suggest that the timescales of the different mechanisms are not substantially different (Maffei et al 2004, Maffei and Turrigiano 2008, Ma et al 2019), we consider this exploration beyond the scope of this paper.

Furthermore, we do agree that studying how systems respond to perturbations when equipped with different combinations of homeostatic mechanisms is a relevant issue. For example, in Páscoa dos Santos et al 2023 we show that large-scale models of the cortex with local homeostasis of inhibition can recover functional connectivity properties following a lesion, but not network dynamics. In a more recent study Páscoa dos Santos and Verschure 2024, we have demonstrated that adding the homeostasis of excitation and excitability homeostasis can help the network recover not only FC but also large-scale dynamics, showcasing the benefits of implementing multiple homeostatic mechanisms. However, in the current manuscript we focus on introducing the mathematical derivation of different modes of E-I homeostasis and studying their impacts circuit node dynamics, particularly oscillations. With that in mind and the fact that these issues have been explored elsewhere, we have chosen to not include this exploration in the current manuscript, which is already heavy with information, to avoid overburdening the reader.

Q2.4 - *Critical slowing down: Making connection with the last point, it would be interesting to explore how the system's ability to recover from a perturbation changes, approaching the bifurcation line from the left, in different homeostasis models. Does the network activity become more correlated in time? Does the effect of noise (additive or multiplicative) become more pronounced? (I know you don't have noise in your model; yet I think exploring how the dynamics is affected by noise would be very interesting.) Scheffer, Marten, et al. "Early-warning signals for critical transitions." Nature 461.7260 (2009): 53-59.*

R2.4 – We thank the reviewer for the suggestion. Following this, we decided to investigate the time it takes for models with different homeostatic mechanisms to recover from perturbations as the bifurcation is approached. To do that, we

simulated the activity of the Wilson-Cowan model with different modes of homeostasis, different values of ρ for 20 seconds and a constant input of $I^{ext} = 2$. To perturb the system, we add a pulse input of magnitude 100 during 10ms at 10s of simulation time and measure the time it takes the system to return to the fixed point. The results are presented in Figure R1. This simple exploration shows that, regardless of the mode of homeostasis implemented, the system always exhibits signatures of critical slowing-down as the bifurcation is approached. While some of the systems show a more pronounced slowing-down (e.g. c^{EI} homeostasis), the difference does not appear significant in this preliminary analysis. With that in mind, and considering that this manuscript is not focused on the recovery of cortical circuits from perturbations through E-I homeostasis (see, for example Páscoa dos Santos et al 2023 or Páscoa dos Santos and Verschure 2024), we believe that this analysis would not add a substantial value to the manuscript as it is. Nonetheless, we believe that it is a relevant topic for further discussion, and have thus added the following paragraph to the discussion.

L624-632: On another note, a relevant phenomenon in edge-of-bifurcation systems that was not studied here is critical slowing-down, relating to the longer time necessary to recover from perturbations in systems close to the bifurcation [73]. While, at the neuronal level, critical slowing-down is thought to provide relevant information about the transition between the quiescent and spiking regimes [74], its effects on brain dynamics at the circuit level are not as clear. Therefore, we suggest that our framework can be used in future studies to study critical slowing down near the Hopf-bifurcation and how it shapes the generation of cortical rhythms.

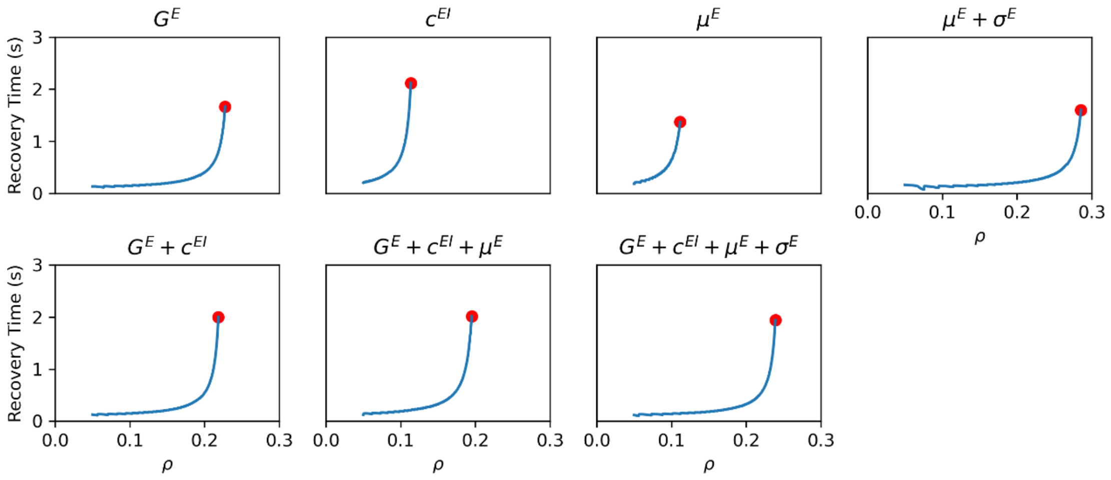

Figure R1 – Critical slowing down in the Wilson-Cowan model with E-I homeostasis. Time to recover from a perturbation (10ms pulse with magnitude 100 a.u.) for different values of the target firing rate (ρ) as the model approaches the supercritical Hopf-bifurcation (red dot). Each subplot represents a model with a different combination of homeostatic mechanisms. Note how, for all models, the recovery time increases substantially close to the bifurcation, which is a signature of critical slowing-down.

Q2.5 - *Adding multiple mechanisms or components to a system can make its behaviour less sensitive to parameter changes (Transtrum, Mark K., et al. "Perspective: Sloppiness*

and emergent theories in physics, biology, and beyond." The Journal of chemical physics 143.1 (2015)). The study's findings support this idea: with multiple homeostasis modes, the range of oscillation frequency becomes narrower. This means the system's behaviour doesn't effectively constrain the value of a single parameter. In other words, the complex model could be simplified. While abstract, mentioning that the full model's behaviour suggests a possible simpler description would enrich the paper and encourage further exploration. In this respect, the Discussion section is thorough but could benefit from some broader perspectives to enhance clarity and stimulate deeper scientific discourse.

R2.5 – Indeed, the range of frequencies is constrained when implementing multiple homeostatic mechanisms, which could be interpreted as the model being less sensitive to parameter changes. However, there are two things to be pointed out. First, one of the consequences of combining multiple homeostatic mechanisms is the smaller variation in parameters (see S3 Fig). Therefore, it is difficult to conclude whether the narrowing of the frequency range is a consequence of the fact that the parameters vary less to maintain E-I homeostasis or of the inherent sloppiness of complexifying the system, which renders it less sensitive to parameter changes. Second, the circuit dynamics relative to the bifurcation are still strongly affected by changes in the parameters modulated by homeostasis (see Fig 5), as opposed to what happens, for example, with μ^E homeostasis (Fig 4c). Therefore, we believe that a simpler model, such as the Stuart-Landau model where the distance to the bifurcation can be directly modulated without changing the frequency, would still be inadequate to reproduce the impact of E-I homeostasis on circuit dynamics. Finally, we also interpret the fact that the range of frequencies is more constrained as a positive effect of employing multiple homeostatic mechanisms, allowing gamma rhythms to remain within physiological bounds.

Minor Issues:

Q2.6 - *The paper is generally well-written and organized. However, there are a few instances where the text could be improved. Just a few suggestions:*

R2.6 – We thank the reviewer for their suggestions on the text and have thus made improvements to the referred sections.

Abstract: Therefore, we investigate how the diverse mechanisms of E-I homeostasis employed by cortical networks shape oscillations and edge-of-bifurcation dynamics

L383: Notably, homeostasis no longer requires

L604-606: In this context, research suggests that optimal cortical dynamics occur when nodes have heterogeneous distances to the bifurcation

Comments from Reviewer 3

The authors investigate the homeostatic control of firing rates in a model network by a combination of analytical and numerical methods.
In the present study there are 2 novelties with respect to the existing studies: (i) the authors consider the operation of multiple, experimentally-demonstrated, mechanisms of homeostasis (i.e., synaptic scaling of E-to-E and I-to-E connections, and plasticity of intrinsic excitability) both in isolation and in combination; (ii) the authors investigate the 'side effects' of the homeostatic control of the firing rates, that is, how the distance from the bifurcation (between a stable fixed-point and a limit cycle) and the frequency of the oscillations (damped and sustained) are impacted by the different homeostatic mechanisms.
The main result is a proof-of-concept that the distance to criticality (here, distance from the fixed-point-to-limit-cycle bifurcation) can be, indeed, regulated by properly (i.e., by the right combination of homeostatic mechanisms) controlling the firing rates. This is obviously relevant for theories of brain criticality (in a broad sense). The results presented also suggest some of the potential mechanisms by which regional specialization (at the large-scale level) could be obtained starting from 'homogeneous' building blocks (i.e., the local networks).
The study is carefully carried-out, the results are novel and certainly interesting, the manuscript is well written. I have only a few, rather minor, comments.
There are several simplifications/assumptions in the modeling. That is perfectly fine, modeling is essentially about simplifying a problem. However, the potential impact of the simplifications on the results should be clearly discussed.

Q3.1 - *For instance, the authors point out that in the original model (lines 104-112) the inhibitory population is self-coupled (i.e., inhibitory neurons inhibit themselves besides inhibiting the excitatory neurons). To be sure, inhibitory self-coupling at the population level is also demonstrated experimentally. In the manuscript, they set this coupling to zero, because otherwise the model does not display the bifurcation. However, one could be tempted to argue that, then, perhaps this is not the right model, given that to obtain the behavior of interest I have to neglect well-established experimental information. Similarly, the inhibitory population does not receive external inputs in the model, while it certainly does in the cortex. More generally, to which extent the results depend on the choice of the default parameters (Table 1)? Or alternatively, what is the rationale for this specific choice? The authors only provide a rationale for the choice of the time constants. For instance, inhibitory neurons fire at a much higher rate than excitatory neurons in the cortex.*

> **R3.1 –** We thank the reviewer for this insightful comment. Indeed, it is true that self-inhibitory coupling has been demonstrated experimentally, not only between interneuron types, but also between fast-spiking interneurons, which are the focus of our study (Potjans and Disemann 2012, Tremblay 2016). In addition, we assume that there is no external input to the inhibitory population, based on the fact long-range coupling in large-scale neural mass models of the cortex is generally implemented between excitatory populations only. However, experimental results show that cortical interneurons receive similar input patterns to their pyramidal counterparts (Brown et al 2020, Okoro et al 2022).

In this work, we made both assumptions, in line with previous studies [cite], to simplify the equations and the analytical solution of the system. Nonetheless, it is still possible to follow the same rationale and methodology to analyze circuits with both I-I coupling and external input to the inhibitory population. Therefore, to answer the reviewer's concern, we have analyzed the dynamics of the Wilson-Cowan model with homeostasis of excitation, inhibition, and intrinsic excitability and different levels of self-inhibition (c^{II}) and external input to the inhibitory population (I_I^{ext}). Since results demonstrate that the inputs to E and I populations in layer 2/3 receive strongly correlated inputs from distant areas (Brown et al 2020, Okoro et al 2022), we write $I_I^{ext} = \alpha I^{ext}$ and analyze the results as a function of α, the ratio between the input excitatory and inhibitory populations receive. We thus present the results in Figure R2.
In short, our analysis suggests that while avoiding the c^{II} and I_I^{ext} simplifies the analysis and is a direct way of guaranteeing that a Hopf-bifurcation is possible, this dynamical behavior can still exist under different combinations of both parameters. More specifically, if both values are below a certain threshold, the system can still undergo a supercritical Hopf bifurcation depending on the parameter ρ and the bifurcation portrait is qualitatively similar to the one obtained for our simplification. Furthermore, we have observed an interaction between c^{II} and I_I^{ext}, indicating that the loss of the Hopf-bifurcation due to increased input to the inhibitory population can be avoided by increasing the self-inhibitory coupling c^{II}. However, if self-inhibition becomes too high (e.g. $c^{II} = 4$), the model can no longer exhibit the Hopf-bifurcation. Therefore, we believe that our results are still compatible with the architecture of cortical networks, provided that self-inhibition is weaker compared, for example, to I-E coupling and that external inputs are weaker in inhibitory neurons than in their excitatory counterparts (for example, a circuit with $c^{II} = 1$ and $\alpha = 0.25$). More importantly, both constraints are justified to a certain degree given the literature on the organization of cortical circuits (**weaker self-inhibition**: Potjans and Diesmann 2012 and Hage et al 2022 ; **weaker input to inhibitory neurons**: Table 4, Potjans and Diesmann 2012).

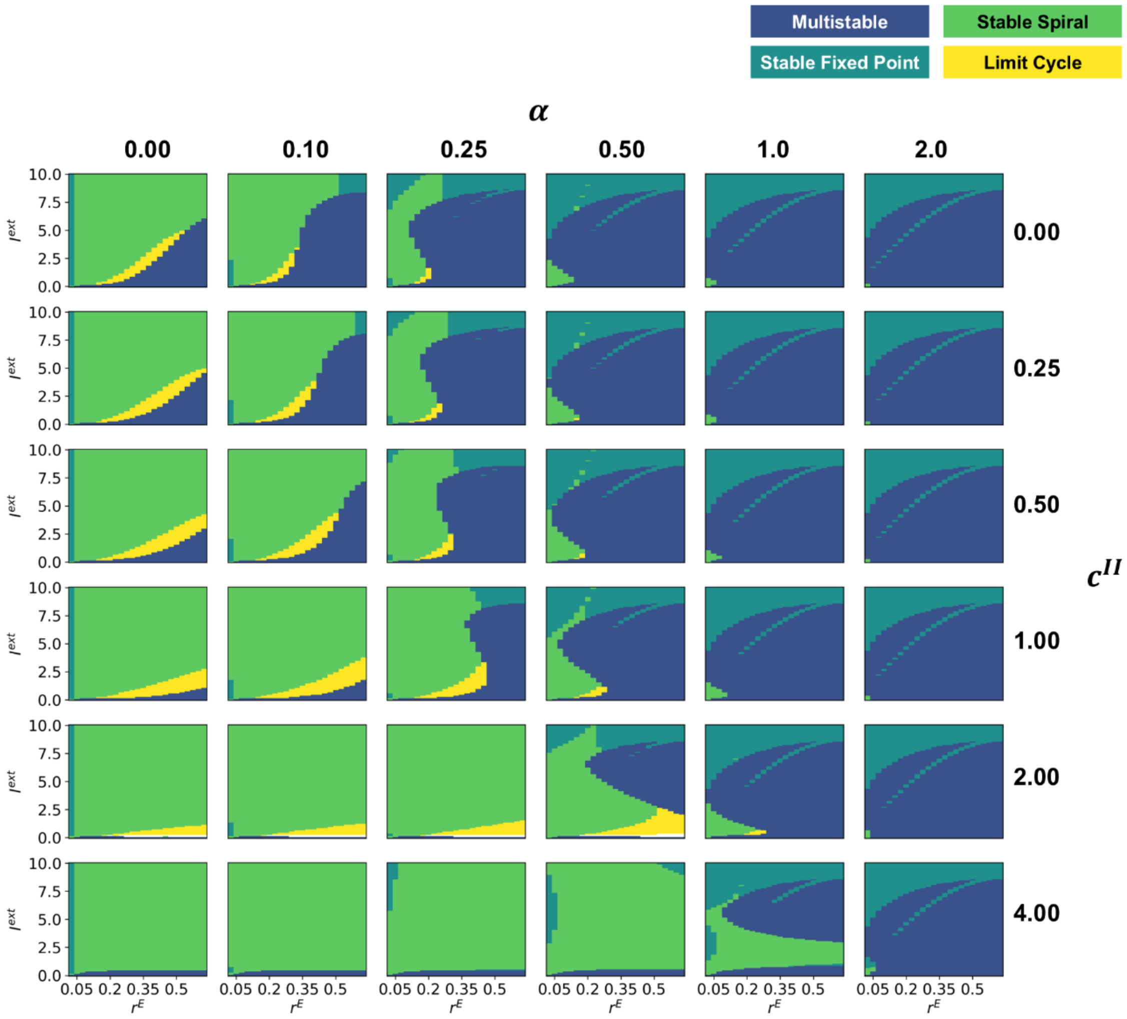

Figure R2 - Circuit dynamics in models with different combinations of c^{II} and α. Circuit dynamics of models under homeostasis of G^E, c^{EI}, μ^E, and σ^E with different combinations of external input I^{ext} and fixed point r^E. Colors represent the result of linear stability analysis for each combination of parameters, as described by the legend (top-left). In each sub-plot, we present the results of this analysis for models with a different combination of c^{II}, the strength of self-inhibition, and α, representing the relative strength of the external input to the inhibitory population, compared to I^{ext} (input to the excitatory neural mass).

Given these new considerations, we have added Figure R2 to the supplementary material with a more detailed description of the results. In addition, we have updated the methods section to specify that, while the assumptions of $c^{II} = 0$ and $I_I^{ext} = 0$ were made, the behavior of the model was also explored with different combinations of these parameters.

L111-117: Nonetheless, we explore the effects of E-I homeostasis in models with different levels of self-inhibition (c^II) (S1 Appendix). In addition, we also study the behavior of models under different levels of input to the inhibitory population (S1 Appendix). Importantly, while we set both these parameters to 0 in the main text, the Hopf-bifurcation can still exist in circuits with self-inhibition and input to the inhibitory population, provided that both these values are kept within certain bounds. For a more detailed explanation, refer to S1 Appendix.

Regarding the fact that inhibitory neurons fire at a much higher rate than their excitatory counterparts, we point out that this can be easily accounted for in the model, by adapting the input-out function $F(x)$ into $F(x) = \frac{F_{max}}{1+e^{-\frac{x-\mu}{\sigma}}}$, where F_{max} is the maximum firing rate of a neuronal population. Then, the model can be made to exhibit the same dynamics by adjusting the other parameters accordingly. For example, if F_{max} in the inhibitory population is increased to from 1 to 2.5, the model exhibits the same behavior if c^{EI} is decreased from 2.5 to 1 (see Figure R3). Therefore, we argue that, since the system can be adapted to account for the differences in the magnitude of E and I firing rates in the cortex, our conclusions would still be valid in a model with substantially higher inhibitory firing rates.

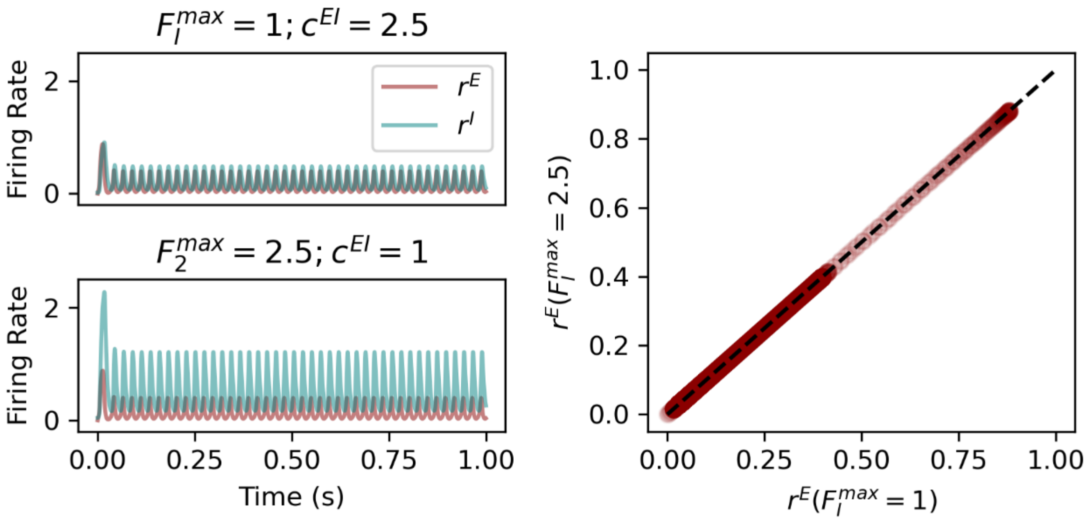

Figure R3 – Comparison between models with different maximum inhibitory firing rates (F_I^{max}). (Top Left) Excitatory and inhibitory activity in a model with $F_I^{max} = 1$ and $c^{EI} = 2.5$. (Bottom Left) Excitatory and inhibitory activity in a model with $F_I^{max} = 2.5$ and $c^{EI} = 1$. For both simulations we have used the same initial conditions and no added noise. (Right) Correlation between excitatory activity in both simulations. Note that activity is perfectly correlated, indicating that the maximum firing rates of the model can be scaled up or down to correspond to empirical values without affecting model dynamics, as long as other model parameters are adjusted accordingly.

Finally, in relation to the impact of the default values on the behavior of the model, as argued in R1.1, the default value of a given parameter does not influence the homeostatic setpoint in systems with single mechanisms of homeostasis. In systems with multiple modes of homeostasis, the default values will indeed change the final setpoint (see the examples in S4 Appendix in the revised manuscript). However, a detailed exploration of the effects of changing the default parameters would require an extensive parameter search or the fitting of the default parameters to data from controlled recordings, for example in cortical slices, where the conditions and external inputs are easier to manipulate. Therefore, we argue that, with its limitations, our study still provides relevant answers to the issue of how bifurcation control can emerge in cortical circuits from E-I homeostasis. Nonetheless, we have updated the discussion section to comment on this limitation (see R1.1).

Q3.2 - *Eqs. (6), (7) and (10) only make sense if G^E, c^{EI} and \mu_E are non-negative. Is this obviously always the case? If so, then it should be explained why this is so, and otherwise it should be explained how the non-negativity of the corresponding quantities is ensured, or where is ensured. For instance, it seems to me that G^E in Eq. (6) becomes negative when r^E goes to zero.*

R3.2 – We thank the reviewer for pointing out this issue. In our study, we follow Dale's principle, which constraints G^E and c^{EI} to non-negative values. Conversely, when implementing the homeostasis of μ^E alone, this parameter can decrease to negative values, given that it represents the firing threshold. In practical terms, negative μ^E would relate to neuronal populations with strong baseline activity (i.e. without external inputs). When homeostasis of μ^E and σ^E are considered synergistically, then we also constrain $\mu^E > 0$, since $\sigma^E = k\mu^E$ and negative values would imply a negative slope of the I-F curve, which is not realistic.

For example, as the reviewer points out, G^E in Eq. (6) becomes negative when r^E goes to zero. Therefore, in Figure 4a), the parameter space is empty for lower values of ρ, representing target-firing rates for which G^E would become negative. That said, we recognize that we do not specify these constraints in the text, and have thus updated the methods section to make that clear:

L164-166: Importantly, following Dale's principle [58], we constrain G^E so that it cannot become lower than 0, which effectively would mean that excitatory synapses become inhibitory.

L174: Similarly to G^E, we constrain c^EI>0 to conform with Dale's principle.

L194-196: In this case, a negative slope σ^E would signify that excitatory inputs decrease population activity, and vice-versa, implying, in practice, a violation of Dale's principle. Therefore, we also apply the constraint that σ^E = Kμ^E > 0.

Q3.3 - *Distinguishing dashed from solid lines in, e.g., Fig.4 was extremely complicated for me.*

R3.3 – We have updated the plots in the revised manuscript to make a clearer distinction between dashed and solid lines.

References

Kujala J, Jung J, Bouvard S, Lecaignard F, Lothe A, Bouet R, et al. Gamma oscillations in V1 are correlated with GABA(A) receptor density: A multi-modal MEG and Flumazenil-PET study. Scientific Reports. 2015;5:16347.

Kujala J, Ciumas C, Jung J, Bouvard S, Lecaignard F, Lothe A, et al. GABAergic inhibition shapes behavior and neural dynamics in human visual working memory. Cerebral Cortex. 2024;34(2):bhad522.

Ma Z, Turrigiano GG, Wessel R, Hengen KB. Cortical Circuit Dynamics Are Homeostatically Tuned to Criticality In Vivo. Neuron. 2019;104(4):655–664.e4.

Maffei A, Nelson SB, Turrigiano GG. Selective reconfiguration of layer 4 visual cortical circuitry by visual deprivation. Nature Neuroscience. 2004;7(12):1353–1359.

Maffei A, Turrigiano GG. Multiple Modes of Network Homeostasis in Visual Cortical Layer 2/3. Journal of Neuroscience. 2008;28(17):4377–4384.

Páscoa dos Santos F, Vohryzek J, Verschure PFMJ. Multiscale effects of excitatory-inhibitory homeostasis in lesioned cortical networks: A computational study. PLOS Computational Biology. 2023;19(7):e1011279.

Páscoa dos Santos F, Verschure PFMJ. Metastable Dynamics Emerge from Local Excitatory-Inhibitory Homeostasis in the Cortex at Rest. bioRxiv. 2024

Turrigiano G. Too many cooks? Intrinsic and synaptic homeostatic mechanisms in cortical circuit refinement. Annual Review of Neuroscience. 2011;34:89–103

Wen W, Turrigiano GG. Keeping Your Brain in Balance: Homeostatic Regulation of Network Function. Annual Review of Neuroscience. 2024;
