# Supplementary material for: Excitatory-inhibitory homeostasis and bifurcation control in the Wilson-Cowan model of cortical dynamics
Source: PLoS Comput Biol. 2025 Jan 6;21(1):e1012723. doi: 10.1371/journal.pcbi.1012723 (PMC11737862; doi:10.1371/journal.pcbi.1012723)
Supplement: Attachment — Submitted filename: Review Guido Gigante.docx [file pcbi.1012723.s016.docx]

This paper investigates the role of excitatory-inhibitory (E-I) homeostasis in shaping the dynamics of cortical networks, particularly focusing on how different homeostatic mechanisms influence oscillations and the system's proximity to a bifurcation point. The authors employ the Wilson-Cowan (WC) model, a classic model of cortical dynamics, and extend it to incorporate various modes of E-I homeostasis. They derive analytical solutions for the fixed points of the system under different homeostatic mechanisms and use bifurcation analysis to characterize the system's behaviour.

The key findings are:

- **E-I homeostasis can control the system's proximity to a bifurcation point**, influencing whether the system exhibits damped oscillations, sustained oscillations, or stable fixed points.
- **Different modes of homeostasis have distinct effects on the bifurcation point**. Some modes shift the bifurcation towards higher firing rates with increased external input, while others shift it towards lower firing rates or keep it independent of input.
- **Multiple modes of homeostasis acting in conjunction can lead to more robust regulation of network dynamics** and keep oscillation frequencies within a physiologically plausible range.
- **The time constant of inhibition also influences the bifurcation point**, with slower inhibition making sustained oscillations more likely.

The most interesting claims, in my view, are those related to the **heterogeneity of bifurcation points across cortical areas** and the **functional benefits of employing multiple homeostatic mechanisms**. These claims provide potential explanations for observed phenomena in the cortex and offer insights into the design principles of cortical networks.

The results presented in this paper are intriguing and potentially significant. However, there are a few points that require further clarification and investigation to strengthen the conclusions.

1. **Generality of the findings**: The study focuses on the WC model, which is a simplified representation of cortical dynamics. While the WC model captures essential features of E-I interactions, it would be important to discuss the extent to which the findings can be generalized to more complex and biologically realistic models of cortical networks.
2. **Novelty of the multiple homeostasis approach**: The authors claim that their approach of considering multiple homeostatic mechanisms acting in conjunction is novel. While this is a valuable contribution, it would be beneficial to provide a more thorough discussion of how this approach differs from previous studies that have also considered multiple homeostatic mechanisms (*e.g.*, Cannon, Jonathan, and Paul Miller. "Synaptic and intrinsic homeostasis cooperate to optimize single neuron response properties and tune integrator circuits." Journal of neurophysiology 116.5 (2016): 2004-2022).
3. **Additional benefits**: The functional benefits of employing multiple homeostatic mechanisms could further be highlighted by, for example:
   - Exploring the effects of different homeostatic timescales on network dynamics and stability.
   - Quantifying the robustness of network dynamics to perturbations under different combinations of homeostatic mechanisms to demonstrate the benefits of multiple homeostasis.
4. **Critical slowing down**: Making connection with the last point, it would be interesting to explore how the system’s ability to recover from a perturbation changes, approaching the bifurcation line from the left, in different homeostasis models. Does the network activity become more correlated in time? Does the effect of noise (additive or multiplicative) become more pronounced? (I know you don’t have noise in your model; yet I think exploring how the dynamics is affected by noise would be very interesting.)
   Scheffer, Marten, et al. "Early-warning signals for critical transitions." Nature 461.7260 (2009): 53-59.
5. **Model becoming more “sloppy”**: Adding multiple mechanisms or components to a system can make its behaviour less sensitive to parameter changes (Transtrum, Mark K., et al. "Perspective: Sloppiness and emergent theories in physics, biology, and beyond." The Journal of chemical physics 143.1 (2015)). The study's findings support this idea: with multiple homeostasis modes, the range of oscillation frequency becomes narrower. This means the system's behaviour doesn't effectively constrain the value of a single parameter. In other words, the complex model could be simplified.
   While abstract, mentioning that the full model's behaviour suggests a possible simpler description would enrich the paper and encourage further exploration.
   In this respect, the Discussion section is thorough but could benefit from some broader perspectives to enhance clarity and stimulate deeper scientific discourse.

**Minor Comments**

The paper is generally well-written and organized. However, there are a few instances where the text could be improved. Just a few suggestions:

- “The first noteworthy result is homeostasis no longer requires” -> “Notably, homeostasis no longer requires”.
- "In this context, some studies suggest that cortical dynamics are optimized when nodes have heterogeneous distances to the bifurcation" -> "In this context, research suggests that optimal cortical dynamics occur when nodes exhibit varying distances to the bifurcation".
- "Therefore, we investigate how oscillations and edge-of-bifurcation dynamics are shaped by the diverse mechanisms of E-I homeostasis employed by cortical networks" -> "Therefore, we investigate how the diverse mechanisms of E-I homeostasis employed by cortical networks shape oscillations and edge-of-bifurcation dynamics".

This paper presents a valuable contribution to the understanding of E-I homeostasis and its role in shaping cortical dynamics. The analytical solutions and bifurcation analysis provide insights into the mechanisms by which E-I homeostasis controls network behaviour. The claims about the heterogeneity of bifurcation points and the benefits of multiple homeostasis are particularly intriguing and have the potential to stimulate further research in this area. However, addressing the points raised above would strengthen the paper and make the conclusions more convincing. I recommend this paper for publication after minor revisions.
